# Hidden-symmetry-enforced nexus points of nodal lines in layer-stacked dielectric photonic crystals

**DOI:** 10.1038/s41377-020-00382-9

**Published:** 2020-10-19

**Authors:** Zhongfei Xiong, Ruo-Yang Zhang, Rui Yu, C. T. Chan, Yuntian Chen

**Affiliations:** 1 School of Optical and Electronic Information, Huazhong University of Science and Technology, Wuhan, 430074 China; 2 Department of Physics, The Hong Kong University of Science and Technology, Clear Water Bay, Hong Kong, China; 3 School of Physics and Technology, Wuhan University, Wuhan, 430072 China; 4 Wuhan National Laboratory of Optoelectronics, Huazhong University of Science and Technology, Wuhan, 430074 China

**Keywords:** Photonic crystals, Optical physics

## Abstract

It was recently demonstrated that the connectivities of bands emerging from zero frequency in dielectric photonic crystals are distinct from their electronic counterparts with the same space groups. We discover that in an AB-layer-stacked photonic crystal composed of anisotropic dielectrics, the unique photonic band connectivity leads to a new kind of symmetry-enforced triply degenerate points at the nexuses of two nodal rings and a Kramers-like nodal line. The emergence and intersection of the line nodes are guaranteed by a generalized 1/4-period screw rotation symmetry of Maxwell’s equations. The bands with a constant *k*_*z*_ and iso-frequency surfaces near a nexus point both disperse as a spin-1 Dirac-like cone, giving rise to exotic transport features of light at the nexus point. We show that spin-1 conical diffraction occurs at the nexus point, which can be used to manipulate the charges of optical vortices. Our work reveals that Maxwell’s equations can have hidden symmetries induced by the fractional periodicity of the material tensor components and hence paves the way to finding novel topological nodal structures unique to photonic systems.

## Photonic crystals: New insights in layers

In some forms of materials called photonic crystals, light can propagate in unexpected ways that offer the prospect of controlling light using topological concepts for both theoretical investigations and also potential new applications. Photonic crystals containing alternating dielectric layers can affect the motion of light in subtle ways through multiple scattering, including the formation of specific energy bands. Researchers in China, led by Che Ting Chan at Hong Kong University of Science and Technology and Yuntian Chen at Huazhong University of Science and Technology, studied a specific type of layered photonic crystals with properties that vary in different directions. They identified unique arrangements of the energy bands, called nexus points, around which new optical phenomena can emerge. Their work offers new insights into the hidden symmetry of Maxwell’s equations, which describe the propagation of light.

## Introduction

Discovering and synthesizing symmetry-protected topological (SPT) band degeneracies, including nodal points^1–13^ and nodal lines (NLs)^14–25^, is a rapidly growing frontier in the field of topological materials. The initial impetus for the area came from realizing elusive relativistic quasi-particles, e.g., 3-dimensional (3D) Weyl and Dirac fermions, in both electronic crystalline materials^1–4^ and photonic crystals (PhCs)^5–13^. Interestingly, since the crystallographic space groups impose fewer constraints on the energy bands than the continuous Poincaré group, more exotic multifold band crossings were found in lattice systems^26^, which have no counterparts in high-energy physics. As a remarkable example, certain space groups allow the existence of triply degenerate points in the band structures, forming either as isolated point nodes carrying monopole charges, so-called spin-1 Weyl points^26–30^, or as nexuses connecting several NLs^31–33^. On the other hand, the SPT band crossings can also be classified according to whether they are merely symmetry-allowed (accidental) or symmetry-enforced^34,35^. The former are only perturbatively stable, whereas the symmetry-enforced degeneracies are robust against any large symmetry-preserving deformations and are currently drawing more attention due to their deterministic nature^25,34,35^.

In PhCs, the topology of band structures is usually thought to be adequately described by spinless space groups, provided that special internal symmetries, such as electromagnetic (EM) duality, are not imposed on the EM materials. However, in dielectric PhCs, there are always two gapless bands emerging from zero frequency and momentum, $\omega = |{\mathbf{k}}| = 0$, irrespective of the space group representations at that point. Watanabe and Lu recently revealed that this intrinsic singularity of EM fields permits higher minimal connectivity for the lowest photonic bands than for their electronic counterparts without spin-orbit coupling and may further enforce unique photonic band crossings even in symmorphic lattices^36^. This pioneering study uncovered the tip of the hidden characteristics of Maxwell’s equations that are relevant to photonic band connectivities. In general, the stationary Maxwell’s equations can be written as a generalized eigenvalue problem,

1 $$\begin{array}{*{20}{c}} {\left( {\begin{array}{*{20}{c}} 0 & {i\nabla \times } \\ { - i\nabla \times } & 0 \end{array}} \right)\left( {\begin{array}{*{20}{c}} {\mathbf{E}} \\ {\mathbf{H}} \end{array}} \right) = \omega \left( {\begin{array}{*{20}{c}} {{\overleftrightarrow{\varepsilon}}\left( {\mathbf{r}} \right)} & {{\overleftrightarrow{\chi}}\left( {\mathbf{r}} \right)} \\ {{\overleftrightarrow{\chi}} ({\mathbf{r}})^\dagger } & {{\overleftrightarrow{\mu}} \left( {\mathbf{r}} \right)} \end{array}} \right)\left( {\begin{array}{*{20}{c}} {\mathbf{E}} \\ {\mathbf{H}} \end{array}} \right)} \end{array}$$

where we henceforth denote the curl matrix and the constitutive matrix on the left and right sides of Eq. (1) as $\widehat {\cal{N}}({\mathbf{r}})$ and $\widehat {\cal{M}}({\mathbf{r}})$, respectively. Since all space group transformations leave the curl matrix $\widehat {\cal{N}}({\mathbf{r}})$ invariant, a PhC respects a space group symmetry $\hat A$ only if its constitutive tensor obeys $\hat A\,\widehat {\cal{M}}({\mathbf{r}})\hat A^{ - 1} = \widehat {\cal{M}}({\mathbf{r}})$. However, a generic symmetry $\tilde A$ of Maxwell’s Eq. (1) operates on the Hamiltonian $\widehat H({\mathbf{r}}) = \widehat {\cal{M}}({\mathbf{r}})^{ - 1}\widehat {\cal{N}}({\mathbf{r}})$ of EM fields, namely, requiring $\tilde A \widehat H\left( {\mathbf{r}} \right)\tilde A^{ - 1} = \widehat H({\mathbf{r}})$, and not on $\widehat {\cal{N}}({\mathbf{r}})$ and $\widehat {\cal{M}}({\mathbf{r}})$ separately. This fact implies that the conventional space groups alone are insufficient to determine the symmetry properties as well as the band connectivities of photonic systems.

In this work, we propose a simple layer-stacked photonic structure consisting of anisotropic dielectrics to exemplify such hidden symmetries of Maxwell’s equations beyond space groups. We show that a hidden symmetry, more specifically, a generalized fractional screw rotation symmetry, together with time reversal symmetry guarantees the emergence of Kramers-like straight NLs passing through the Brillouin zone (BZ) centre and results in unusual photonic band connectivities. Furthermore, we demonstrate that the lowest Kramers-like NL can almost always intersect with two other SPT nodal rings at two triply degenerate nexus points (NPs), which can be seen as a new kind of magnetic monopole connecting Berry flux strings in momentum space^37–39^. By breaking the hidden symmetry, we lift the two NPs and achieve type-II and type-III nodal rings in the PhC^19–21^. In addition, the peculiar anisotropic band structure in the vicinity of the NPs, especially the spin-1 conical dispersion of the iso-frequency surfaces, may lead to novel transport phenomena. As an example, we show how optical vortices can be manipulated via the spin-1 conical diffraction effect^40^ for a beam incident at an NP.

## Results

**Fig. 1:**
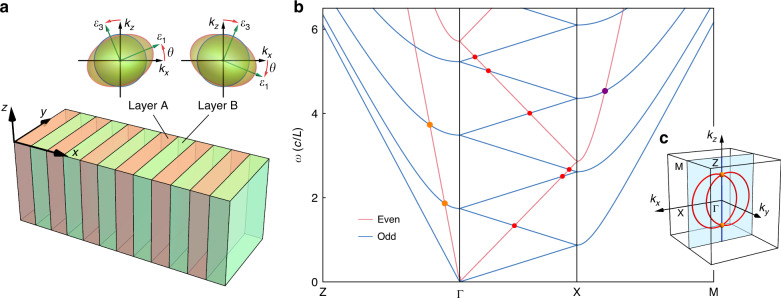
AB-layer-stacked photonic crystal made of a generic biaxial dielectric. **a** Structure of the PhC, where the two insets display the iso-frequency surfaces in the *xz* plane of the biaxial dielectrics in layer A (orange) and layer B (green). **b** Band structure along high symmetry lines in the *k*_*y*_ = 0 plane for the PhC with $\varepsilon _1 = 2,\varepsilon _2 = 13,\varepsilon _3 = 1$, and rotation angle *θ* = *π*/5. The blue and magenta lines represent the bands with odd and even $\widehat M_y$-parities, respectively. The red dots correspond to the nodal lines along which two bands with opposite $\widehat M_y$-parities intersect. The orange and purple dots denote the threefold and fourfold degenerate nexus points, respectively. **c** Sketch map of two red nodal rings (corresponding to the two lowest red dots in (**b**)) crossing a Kramers-like nodal line (blue line) at two nexus points in the first (bulk) Brillouin zone of the PhC

The photonic crystal considered here consists of two types of dielectric layer (A and B) stacked periodically along the *x* direction, which are homogeneous in the transverse *yz* plane. The A and B layers have equal thicknesses, *L*/2, and are both composed of the same kind of nondispersive anisotropic dielectric with principal relative permittivity values of $\varepsilon _1,\varepsilon _2,{\mathrm{and}}\,\varepsilon _3\,( \ne \varepsilon _1)$, whereas the optical axes of the dielectric rotate alternatively in the AB layers, as shown in Fig. 1a. Specifically, the second principal axis of the materials is fixed along the *y* direction, while the first principle axis is rotated by an angle *θ* counterclockwise (clockwise) from the *x*-axis in layer A (B). As such, the PhC’s relative permittivity tensor in *xyz* coordinates is given by

2 $$\begin{array}{*{20}{c}} {{\overleftrightarrow{\varepsilon}}_r = \left( {\begin{array}{*{20}{c}} {\varepsilon _{xx}} & 0 & {\varepsilon _{xz}} \\ 0 & {\varepsilon _{yy}} & 0 \\ {\varepsilon _{zx}} & 0 & {\varepsilon _{zz}} \end{array}} \right)} \end{array}$$

where $\varepsilon _{xz} = \varepsilon _{zx} = \pm g = \pm \left( {\varepsilon _1 - \varepsilon _3} \right){\mathrm{sin}}\theta {\mathrm{cos}}\theta$ flips its sign between layers A and B, while the diagonal elements $\varepsilon _{xx} = \left( {{\mathrm{cos}}^2\theta \varepsilon _1 + \varepsilon _3{\mathrm{sin}}^2\theta } \right)$, *ε*_*yy*_ = *ε*_2_, and $\varepsilon _{zz} = \left( {{\mathrm{sin}}^2\theta \varepsilon _1 + \varepsilon _3{\mathrm{cos}}^2\theta } \right)$ are all constant. The band structure along high symmetry lines of the PhC is plotted in Fig. 1b (see Supplementary Information S1 for the analytical calculation).

### Space group symmetries

The space group of the PhC is ${\Bbb R}^2\rtimes {\mathrm{Rod}}(22)$, i.e., the semidirect product of the two-dimensional continuous translational group ${\Bbb R}^2$ in the *yz* plane and the nonsymmorphic rod group **22** (*pmcm*)^41^ associated with discrete translations along the *x*-axis. Here, we focus on several space group symmetries relevant to the band crossings in the *k*_*y*_ = 0 plane.

First, the PhC is invariant under the mirror reflection ($\widehat M_y$) about the *y* = constant planes, which permits the bands with opposite (odd and even) mirror parities to intersect along NLs in the $\widehat M_y$-invariant plane *k*_*y*_ = 0^14–16^, as marked by the red dots in Fig. 1b and the red rings in Fig. 1c (also see Fig. 3 for the 3D band structures). Second, the combined inversion and time reversal symmetry (${\cal{P}}{\cal{T}}$) quantizes the Berry phase encircling the NLs as *π*, stabilizing the NLs against local ${\cal{P}}{\cal{T}}$-preserving perturbations^15–18^. Third, the PhC has a twofold screw symmetry $\hat S_{2x}:(x,y,z) \to (x + \frac{L}{2}, - y, - z)$. Together with ${\cal{T}}$, the combined symmetry ${\hat{\mathrm \Theta }}_{L/2} = {\cal{T}}\hat S_{2x}$ ensures that all Bloch states are doubly degenerate in the *k*_*x*_ = ±*π*/*L* plane (corresponding to the twofold degenerate bands along X–M in Fig. 1b)^12^.

However, although the space group only supports 1D irreducible representations along the Γ–Z line, the band structure shows that two bands with the same $\widehat M_y$-parity always linearly cross along this line regardless of the dielectric parameters, and accordingly, the two red nodal rings intersect at two NPs (orange dots in Fig. 1c). This indicates that the PhC system possesses a symmetry beyond the crystallographic space group.

### Hidden symmetry and Kramers-like NLs

Since the permittivity ${\overleftrightarrow{\varepsilon}} _r({\mathbf{r}})$ and the Hamiltonian $\widehat H({\mathbf{r}}) = \widehat {\cal{M}}({\mathbf{r}})^{ - 1}\widehat {\cal{M}}$ of EM fields are generically tensors, the periodicity of the system restricts the period of each component of ${\overleftrightarrow{\varepsilon}}_r({\mathbf{r}})$ to a fraction 1/*n* of the full period. As mentioned in the introduction, the space group symmetries of the PhC, e.g., $\hat A$, are entirely encoded in the space-dependent constitutive tensor as $\hat A\,\widehat {\cal{M}}({\mathbf{r}})\hat A^{ - 1} = \widehat {\cal{M}}({\mathbf{r}})$. However, a generic symmetry $\tilde A$ of Maxwell’s Eq. (1) implies that the whole Hamiltonian is invariant under $\tilde A$, i.e., $\tilde A \widehat H\left( {\mathbf{r}} \right)\tilde A^{ - 1} = \widehat H({\mathbf{r}})$, but allows $\tilde A \widehat{\cal{M}}({\mathbf{r}})\tilde A^{ - 1} \ne \widehat{\cal{M}}({\mathbf{r}})$. Here, we show that such hidden symmetry can emerge from the fractional periodicity of the dielectric components in Eq. (2), thereby giving rise to Kramers-like NLs along Γ–Z.

As an accessible entry point, we first consider the ${\widehat M}_y$-odd subsystem in the *k*_*y*_ = 0 plane. Since the PhC is homogeneous in the *y* direction, the $\widehat M_y$-odd eigenstates, $\psi ^{{\mathrm{odd}}} = (E_y,H_x,H_z)^\intercal$, only depend on *ε*_*yy*_. As *ε*_*yy*_ is a global constant for the PhC in Fig. 1, the dispersion along Γ–X can be regarded as simple folding of a linear band, giving rise to degeneracies at Γ and X. Let us consider a relaxed condition $\varepsilon _{yy}(x + L{\mathrm{/}}4) = \varepsilon _{yy}(x)$. In this case, the odd subsystem has a fractional period *L*/4; hence, the width of the BZ of the subsystem is quadrupled in the *x* direction. The $\widehat M_y$-odd band structure in the original BZ can be obtained by folding the bands in the quadruple BZ twice. In the quadruple BZ, the time reversal symmetry ensures that the eigenstates at ±*k*_*x*_ have degenerate eigenfrequencies $\omega (k_x) = \omega ( - k_x)$. After band folding, every pair of eigenstates with identical frequencies at *k*_*x*_ = ±2*π*/*L* is shifted onto the same point along the central line *k*_*x*_ = 0. Consequently, the (4*m* + 2)^th^ and (4*m* + 3)^th^ $\widehat M_y$-odd bands (*m* ≥ 0 is an integer) are degenerate along Γ–Z, as shown in Fig. 1b.

Even though the $\widehat M_y$-even subsystem in the *k*_*y*_ = 0 plane, characterized by the submatrix $$\left( {\begin{array}{*{20}{c}} {\varepsilon _{xx}} & {\varepsilon _{xz}} \\ {\varepsilon _{zx}} & {\varepsilon _{zz}} \end{array}} \right)$$ of ${\overleftrightarrow{\varepsilon}} _r$, has the same primitive period *L* as the whole system, it can be demonstrated that the Hamiltonian of the subsystem with respect to the eigenvector $\psi ^{\prime {\mathrm{even}}} = (D_x,E_z,H_y)^\intercal$ will only depend explicitly on *ε*_*xx*_, *ε*_*zz*_, and $\varepsilon _{xz}^2$ after a local *U*(1) gauge transformation $\widehat U(x,k_z) = {\mathrm{exp}}\left[ {ik_z{\int_0^x} {\frac{{\varepsilon _{xz}(\xi )}}{{\varepsilon _{xx}(\xi )}}} d\xi } \right]$ (see Supplementary Information S2). For the AB-layer-stacked PhC in Fig. 1, *ε*_*xx*_, *ε*_*zz*_, and $\varepsilon _{xz}^2$ are all constant; therefore, the intersections of $\widehat M_y$-even bands along Γ–Z also result from the folding of a linear band. If we relax the constraint on the three parameters from being homogeneous to having a fractional period *L*/*n*, then band crossings along Γ–Z can still exist. In fact, 4 is the minimum value of *n* that maintains the space group ${\Bbb R}^2\rtimes {\mathrm{Rod}}(22)$ of the layer-stacked PhC and preserves the appearance of the NLs along Γ–Z. More specifically, the elements of the permittivity tensor should satisfy

3 $$\begin{array}{*{20}{c}} {\varepsilon _{ii}\left( {x + L{\mathrm{/}}4} \right) = \varepsilon _{ii}\left( x \right)\quad \left( {i = x,y,z} \right)} \end{array}$$

4 $$\begin{array}{*{20}{c}} {\varepsilon _{xz}(x + L{\mathrm{/}}4)^2 = \varepsilon _{xz}(x)^2\quad {\mathrm{and}}\quad \varepsilon _{xz}(x + L{\mathrm{/}}2) = - \varepsilon _{xz}\left( x \right)} \end{array}$$

where the second requirement in Eq. (4) is necessary to keep the primitive period of the PhC of *L*.

If we focus on the minimum requirement case of *L*/4, we can introduce a generalized 1/4-period twofold screw operator and prove that the complete Hamiltonian of both $\widehat M_y$-even and -odd subsystems in the *k*_*y*_ = 0 plane is invariant under the generalized 1/4-period twofold screw rotation (see Supplementary Information S2),

5 $$\begin{array}{*{20}{c}} {\tilde S_{L/4}\hat H\left( {k_y = 0} \right)\tilde S_{L/4}^{ - 1} = \hat H\left( {k_y = 0} \right)} \end{array}$$

**Fig. 2:**
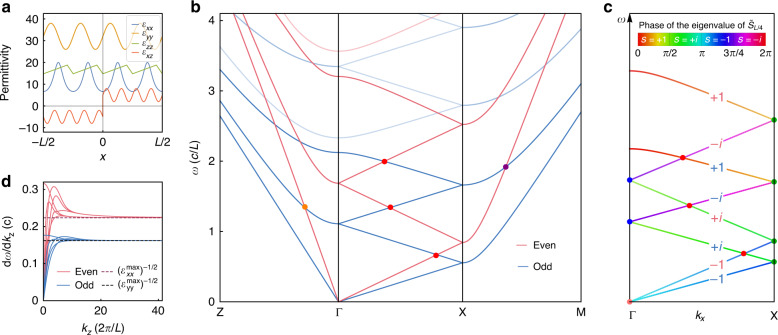
Consequence of the hidden symmetry $\tilde S_{L/4}$ for band connectivity. **a** Profiles of the nonzero permittivity components in one period for a PhC obeying Eqs. (3) and (4). **b** Band structure along high symmetry lines for the PhC with the permittivity given by (**a)**. **c** Connectivity of the lowest four $\widehat M_y$-even and lowest four $\widehat M_y$-odd bands along Γ–X. The colour at a point on the bands displays the phase of the eigenvalue of $\tilde S_{L/4}$ at that point. The labels ±1, ±*i* denote the branch indices, *s*, of the bands. **d** Group velocities ${\mathrm{d}}\omega {\mathrm{/d}}k_z$ of the even (magenta) and odd (blue) bands changing along Γ–Z, which converge to the asymptotic values $c{\mathrm{/}}\sqrt {\varepsilon _{xx}^{{\mathrm{max}}}}$and $c{\mathrm{/}}\sqrt {\varepsilon _{yy}^{{\mathrm{max}}}}$ (two dashed lines), respectively, as *k*_*z*_ → ∞

The generalized 1/4-period twofold screw rotation about the *x*-axis is defined as

6 $$\begin{array}{*{20}{c}} {\tilde S_{L/4} = \left( {\hat P_ - + \hat G^{ - 1}\hat U^\dagger \hat P_ + } \right)\hat C_{2x}\hat T_x\left( {\frac{L}{4}} \right)\left( {\hat P_ - + \hat G\hat U\hat P_ + } \right)} \end{array}$$

where $\hat C_{2x}$ is the twofold rotation about the *x*-axis, $\hat T_x(\frac{L}{4})$ denotes the 1/4-period translation along the *x* direction, $\hat P_ \pm = \frac{1}{2}(\hat I_{6 \times 6} \pm \widehat M_y)$ are the projection operators onto $\widehat M_y$-even/odd subsystems, $\widehat U$ is the aforementioned local *U*(1) gauge transformation, and

7 $$\begin{array}{*{20}{c}} {\hat G = \hat I_{6 \times 6} + \left( {\varepsilon _{xx}\left( x \right) - 1} \right)\hat {\mathbf{e}}_1\hat {\mathbf{e}}_1 + \varepsilon _{xz}\left( x \right)\hat {\mathbf{e}}_1\hat {\mathbf{e}}_3} \end{array}$$

transforms the eigenvector from ${\mathrm{\Psi }} = ({\mathbf{E}},{\mathbf{H}})^\intercal$ to ${\mathrm{\Psi }}^\prime = (D_x,E_y,E_z,{\mathbf{H}})^\intercal$ with the basis $(\hat {\mathbf{e}}_i)_j = \delta _{ij}(i,j = 1, \cdots ,6)$. In Fig. 2, we illustrate a more general example of PhCs satisfying Eqs. (3) and (4). The corresponding profiles of the dielectric components and the photonic band structure are shown in Fig. 2a, b, respectively.

The generalized 1/4-period screw rotation operator is pseudo-unitary^42^ and obeys $(\tilde S_{L/4})^4 = \hat T_x(L)$; thus, its eigenvalue for a Bloch state ${\mathrm{\Psi }}(k_x,0,0)$ on the *k*_*x*_-axis should be a fourth root of $e^{ik_xL}$: $\tilde S_{L/4}{\mathrm{\Psi }}^{(s)}(k_x,0,0) = se^{i\,\frac{{k_xL}}{4}}{\mathrm{\Psi }}^{(s)}(k_x,0,0)$, where the branch index *s* = ±1, ±*i* classifies the bands in the *k*_*y*_ = 0 plane into four groups. Combining time reversal symmetry ${\cal{T}}$ with $\tilde S_{L/4}$, we obtain the pseudo-antiunitary symmetry of the PhC ${\hat{\mathrm \Theta }}_{L/4} = {\cal{T}}\,\tilde S_{L/4}$ and have (see Supplementary Information S3)

8 $$\begin{array}{*{20}{c}} {{\hat{\mathrm \Theta }}_{L/4}^2{\mathrm{\Psi }}^{\left( s \right)}\left( {0,0,k_z} \right) = s^2\,{\mathrm{\Psi }}^{\left( s \right)}\left( {0,0,k_z} \right)} \end{array}$$

for the Bloch states on the *k*_*x*_ = *k*_*y*_ = 0 line (Γ–Z). Following a similar derivation as the Kramers theorem, Eq. (8) ensures that when *s* = ±*i*, ${\mathrm{\Psi }}^{( \pm i)}(0,0,k_z)$ and ${\widetilde{\mathrm \Psi }}(0,0,k_z) = {\hat{\mathrm \Theta }}_{L/4}{\mathrm{\Psi }}^{( \pm i)}(0,0,k_z)$ are two distinct Bloch states degenerate along Γ–Z, forming Kramers-like NLs. Furthermore, since $\tilde S_{L/4}{\widetilde{\mathrm \Psi }}(0,0,0) = s^ \ast \,{\widetilde{\mathrm \Psi }}(0,0,0) = \mp i\,{\widetilde{\mathrm \Psi }}(0,0,0)$, each band of *s* = +*i* along the *k*_*x*_-axis has to intersect with a band of *s* = −*i* at Γ (i.e., the blue dots in Fig. 2c).

At BZ boundaries ${\mathbf{k}} = (\! \pm \!\frac{\pi }{L},0,0)$, the combined symmetry ${\hat{\mathrm \Theta }}_{L/2} = {\cal{T}}\hat S_{2x}$ guarantees that an arbitrary state ${\mathrm{\Psi }}^{(s)}(\! \pm \!\frac{\pi }{L},0,0)$ with branch index *s* is degenerate with ${\widetilde{\mathrm \Psi }}(\! \pm \!\frac{\pi }{L},0,0) = {\hat{\mathrm \Theta }}_{L/2}{\mathrm{\Psi }}^{(s)}(\! \pm \!\frac{\pi }{L},0,0)$. Meanwhile, it can be proven that $\tilde S_{L/4}{\widetilde{\mathrm \Psi }}(\! \pm \!\frac{\pi }{L},0,0) = - i\,s^ \ast \,{\widetilde{\mathrm \Psi }}(\! \pm \!\frac{\pi }{L},0,0)$; therefore, every pair of bands intersecting at the zone boundaries $k_x = \pm \!\frac{\pi }{L}$ must have indices of either *s* = +1 and −*i* or *s* = −1 and +*i* (see the pairs of bands connecting at the green dots in Fig. 2c).

Figure 2b exhibits an important difference of the PhC with reduced constraints compared to the PhC consisting of homogeneous layers in Fig. 1: the fourth and fifth $\widehat M_y$-odd (even) bands are gapped along Γ–Z. In fact, since the fourth $\widehat M_y$-odd (even) band has branch index *s* = +1, Eq. (8) indicates that Ψ^(+1)^ and ${\widetilde{\mathrm \Psi }} = {\hat{\mathrm \Theta }}_{L/4}{\mathrm{\Psi }}^{( + 1)}$ can be the same state. To achieve higher band connectivity along Γ–X, we need the components of the permittivity to have a smaller fractional period *L*/*n* (*n* > 4). The layered PhC in Fig. 1 can be viewed as the limiting case of infinitesimal fractional periodicity (*n* → ∞).

### Photonic band connectivity

Dielectric PhCs have a universal feature in that there are always two gapless photonic bands emerging from the singular point $\omega = |{\mathbf{k}}| = 0$, around which the Bloch modes on the two gapless bands are transverse plane waves in the long-wavelength limit. If the PhCs further meet the conditions of Eqs. (3) and (4), then the eigenvalues of $\tilde S_{L/4}$ for the two lowest bands connected to zero frequency are both equal to $- e^{i\,\frac{{k_xL}}{4}}$, namely, the first $\hat M_y$-even and -odd bands have the same branch index *s* = −1 (see Supplementary Information S4). Starting from the first $\hat M_y$-even (odd) band along Γ–X, $\tilde S_{L/4}$ symmetry ensures that at least four bands with branch indices $- 1 \to + i \to - i \to + 1$ (counting from the bottom) concatenate successively at the Kramers-like degeneracies at $k_x = \frac{\pi }{L}$ and *k*_*x*_ = 0, as shown in Fig. 2c. Consequently, the minimal band connectivity (MBC) along Γ–X is 8 for bands connected to zero frequency, which is beyond the prediction (MBC = 4) made by only considering the twofold screw symmetry $\hat S_{2x}$^36^. MBC = 8 implies that the lowest four even and lowest four odd bands inevitably intersect at least three times (the red dots in Fig. 2b, c and in 1b) along the line segment Γ−X; therefore, the unique photonic band connectivity enforces the emergence of the two red nodal rings shown in Fig. 1c. For bands not connected to zero frequency, $\tilde S_{L/4}$ symmetry leads to MBC = 4 along Γ–X, which is also twice the result determined solely by the space group.

In addition, Fig. 2b shows that all bands along Γ–Z tend towards linear dispersion as *k*_*z*_ → ∞. Indeed, it can be rigorously proven that in an $\widehat M_y$-symmetric dielectric PhC with continuous translational symmetry along the *z*-axis, all $\widehat M_y$-even and all $\widehat M_y$-odd bands have identical asymptotic group velocities in the *z* direction, respectively (see details in Supplementary Information S5),

9 $$\begin{array}{*{20}{c}}\displaystyle {\mathop {{{\mathrm{lim}}}}\limits_{k_z \,\to\, \infty } \frac{{d\omega ^{{\mathrm{even}}}}}{{\mathrm{d}}k_z} = \frac{c}{{\sqrt {\varepsilon _{xx}^{{\mathrm{max}}}} }},\ \mathop {{{\mathrm{lim}}}}\limits_{k_z \,\to\, \infty } \frac{{\mathrm{d}\omega ^{{\mathrm{odd}}}}}{{\mathrm{d}}k_z} = \frac{c}{{\sqrt {\varepsilon _{yy}^{{\mathrm{max}}}} }}} \end{array}$$

where $\varepsilon _{ii}^{{\mathrm{max}}}$ denotes the maximum value of *ε*_*ii*_ (*i* = *x*, *y*) in the PhC, and *c* is the speed of light in vacuum, as demonstrated by the numerical results in Fig. 2d. This result can be understood from the physical picture that the EM fields tend to concentrate in the regions of high refractive index. In the short-wavelength limit (*k*_*z*_ → ∞), all the fields will be localized at the maximal permittivity positions, and hence, only $\varepsilon _{xx}^{{\mathrm{max}}}$ and $\varepsilon _{yy}^{{\mathrm{max}}}$ determine the asymptotic dispersion. This special property indicates that as long as $\varepsilon _{xx}^{{\mathrm{max}}}\, \ne \,\varepsilon _{yy}^{{\mathrm{max}}}$, e.g., $\varepsilon _{xx}^{{\mathrm{max}}} \,< \,\varepsilon _{yy}^{{\mathrm{max}}}(\varepsilon _{xx}^{{\mathrm{max}}} \,>\, \varepsilon _{yy}^{{\mathrm{max}}})$, the first $\widehat M_y$-even (odd) band has to intersect with the second and third $\hat M_y$-odd (even) bands, i.e., the first $\hat M_y$-odd (even) Kramers NL, on Γ–Z (the orange dot in Fig. 2b) owing to their different asymptotic group velocities.

As a notable consequence, almost any layer-stacked dielectric PhC respecting $\widehat M_y$ and the hidden symmetry $\tilde S_{L/4}$ (e.g., the AB-layer-stacked PhC in Fig. 1) must carry a pair of triply degenerate nodes as the nexuses of the two $\widehat M_y$-symmetry-protected nodal rings (red rings in Fig. 1c) and first Kramers-like NL (blue line in Fig. 1c) for the bands connecting to $\omega = |{\mathbf{k}}| = 0$, as displayed in Fig. 1c. More examples with different parameters of the PhC are given in Supplementary Information S6, where we can see that the NPs always appear unless the asymptotic group velocities of even and odd bands are accidentally identical, namely, $\varepsilon _{xx}^{{\mathrm{max}}} = \varepsilon _{yy}^{{\mathrm{max}}}$. Nevertheless, these exceptions only form a subset of measure zero for all possible parameters of the PhCs.

We are also aware that the linear asymptotic dispersion along the *z* direction causes infinitely many bands with opposite mirror parities to intersect as long as $\varepsilon _{xx}^{{\mathrm{max}}} \,\ne \,\varepsilon _{yy}^{{\mathrm{max}}}$, hence forming not only infinitely many threefold NPs but also infinitely many fourfold NPs (see the purple dots in Fig. 2c and Supplementary Information S6 for the typical dispersion around a fourfold NP). Hereinafter, we will focus on the lowest pair of triply degenerate NPs in the AB-layer-stacked PhC shown in Fig. 1 and investigate the band dispersion near the NPs.

### Triply degenerate NPs

**Fig. 3:**
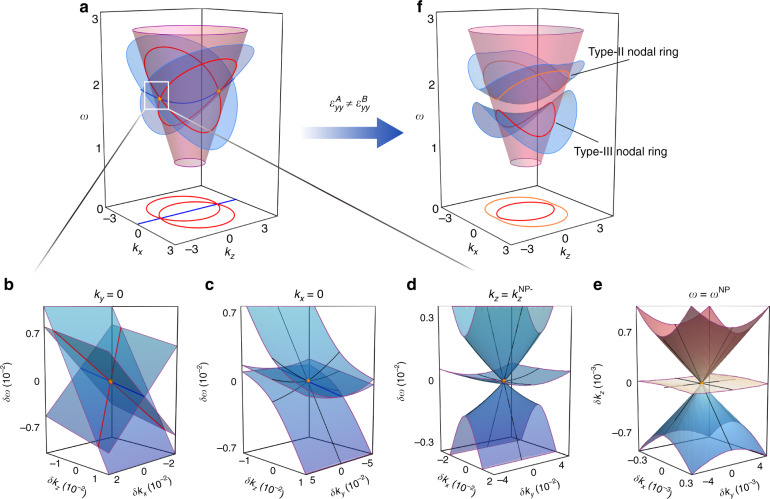
Dispersion near the triple nexus points and nodal lines. **a** 3D band structure of the PhC in Fig. 1 on the high symmetry plane *k*_*y*_ = 0 illustrating that the first $\widehat M_y$-even band (magenta cone) intersects with a pair of $\widehat M_y$-odd bands (light blue surfaces) along two red nodal rings, and the two odd bands coincide along the blue Kramers-like NL. The pair of orange dots shows the nexus points of the 3 NLs. **b**–**d** Zoomed-in band structures around the NP at ${\mathbf{k}}^{{\mathrm{NP}}_ - }$in the *k*_*y*_ = 0, *k*_*x*_ = 0, and $k_z = k_z^{{\mathrm{NP}}_ - }$ sections, respectively, where the vertical coordinate $\delta \omega = \omega - \omega ^{{\mathrm{NP}}}$. **e** Iso-frequency surfaces around the NP at frequency *ω*^NP^, where the black tangential lines are obtained from the first-order **k** · **p** Hamiltonian. **f** Band structure of a PhC with broken hidden symmetry, where *ε*_*yy*_ takes different values in layers A and B of $\varepsilon _{yy}^A = 9$ and $\varepsilon _{yy}^B = 21$, while all the other components of ${\overleftrightarrow{\varepsilon}} _r$ are identical to the case in (**a**)

Figure 3a displays the 3D band structure near the three NLs in the *k*_*y*_ = 0 plane corresponding to the PhC in Fig. 1, where the magenta cone, denoting the first $\widehat M_y$-even band, cuts across the second and third $\widehat M_y$-odd bands (two light blue surfaces) along the two nodal rings. Meanwhile, the two odd bands connect at the Kramers-like NL along *k*_*x*_ = 0. As a result, the three NLs intersect at a pair of triple NPs (orange dots) with Bloch wave vectors ${\mathbf{k}}^{{\mathrm{NP}}_ \pm } = (0,0, \pm \frac{{2\pi }}{L}\sqrt {\frac{{\varepsilon _{xx}}}{{\varepsilon _{yy} - \varepsilon _{xx}}}} )$ and frequency $\omega ^{{\mathrm{NP}}} = \frac{{2\pi c}}{{L\sqrt {\varepsilon _{yy} - \varepsilon _{xx}} }}$. In terms of the **k** · **p** perturbation approach, the effective Hamiltonian around the NPs up to the linear order of $\delta {\mathbf{k}} = (\delta k_x,\delta k_y,\delta k_z) = {\mathbf{k}} - {\mathbf{k}}^{{\mathrm{NP}}_ \pm }$ is given by

10 $$\begin{array}{c}\hat H_{{\mathrm{NP}}}^ \pm = \left( {\begin{array}{*{20}{c}} {v_x\delta k_x \pm v_z^{{\mathrm{odd}}}\delta k_z} & {\frac{{ - i\,v_y}}{{\sqrt 2 }}\delta k_y} & 0 \\ {\frac{{i\,v_y}}{{\sqrt 2 }}\delta k_y} & { \pm v_z^{{\mathrm{even}}}\delta k_z} & {\frac{{ - i\,v_y}}{{\sqrt 2 }}\delta k_y} \\ 0 & {\frac{{i\,v_y}}{{\sqrt 2 }}\delta k_y} & { - v_x\delta k_x \pm v_z^{{\mathrm{odd}}}\delta k_z} \end{array}} \right)\\ = v_x\hat S_z\delta k_x + v_y\hat S_y\delta k_y \pm \left[ {q_z\hat Q_{zz} + v_{z0}\hat I} \right]\delta k_z\end{array}$$

where *v*_*x*_, *v*_*y*_, $v_z^{{\mathrm{odd}}}$, and $v_z^{{\mathrm{even}}}$ are the group velocities along the corresponding directions, $\hat S_i$ (*i* = *x*, *y*, *z*) denote the spin-1 operators, $\hat Q_{zz} = (\hat S_z)^2 - \mathop {\sum}\nolimits_i {(\hat S_i)^2} {\mathrm{/}}3$ is one of the spin-1 quadrupolar operators^28^, $q_z = v_z^{{\mathrm{odd}}} - v_z^{{\mathrm{even}}}$, and $v_{z0} = \frac{1}{3}(2v_z^{{\mathrm{odd}}} + v_z^{{\mathrm{even}}})$ (see Supplementary Information S7 for details).

The bands around NPs present unusual anisotropic dispersion, as depicted by the band structures on different sections passing through an NP (${\mathbf{k}}^{{\mathrm{NP}}_ - }$) in Fig. 3b–d. In the *k*_*y*_ = 0 section, Fig. 3b shows that all three bands linearly cross along the NLs. In Fig. 3c, the band structure in the *k*_*x*_ = 0 section resembles the dispersion of the so-called type-II triply degenerate point^32^, where two bands sandwich and contact the third band along the Kramers-like NL, and all their group velocities ($- v_z^{{\mathrm{odd}}}$ for the NL and $- v_z^{{\mathrm{even}}}$ for the singlet) are of the same sign in the *z* direction. Remarkably, in the $k_z = k_z^{{\mathrm{NP}}_ - } = - \frac{{2\pi }}{L}\sqrt {\frac{{\varepsilon _{xx}}}{{\varepsilon _{yy} - \varepsilon _{xx}}}}$ section, Fig. 3d shows that two conical bands intersect with an almost flat band at the NP, manifesting them as a 2D anisotropic Dirac-like cone described by the 2D spin-1 Hamiltonian $\hat H_{{\mathrm{NP}}}(\delta k_z = 0) = v_x\hat S_z\delta k_x + v_y\hat S_y\delta k_y$^43,44^. Furthermore, as shown in Fig. 3e, the iso-frequency surfaces around the NP at *ω*_NP_ also disperse exactly as a 2D spin-1 Dirac-like cone in the *xy* plane if *δk*_*z*_ is regarded as a pseudo-frequency. This property implies that the NPs in layer-stacked PhCs can be used to realize the novel physical effects associated with 2D spin-1 dispersion.

The unique band topology of the NPs in our system demonstrates that they belong to a new kind of threefold nodal point, different from all isolated triple points carrying topological charge^26–30^. In fact, the charge of an NP cannot be defined because, for an arbitrary closed surface enclosing the NP, the gap between any two of the three bands on the surface must shut at the points where the NLs between the two bands pierce the surface^31,32^. Nonetheless, since the Berry fluxes are confined on the NLs in a ${\cal{P}}{\cal{T}}$ symmetric system, the NPs are the terminations of Berry flux strings and can thus be regarded as a different kind of magnetic monopole, other than Weyl points, in momentum space^37–39^.

### Type-II and type-III nodal rings

The simple AB-layer PhC can be the parent structure of other fancy topological features. In Fig. 3f, we let $\varepsilon _{yy}^A \,\ne \,\varepsilon _{yy}^B$ to observe the process of hidden symmetry $\tilde S_{L/4}$ breaking. Without protection by ${\hat{\mathrm \Theta }}_{L/4} = {\cal{T}}\,\tilde S_{L/4}$, the Kramers-like degeneracy along *k*_*x*_ = 0 between the second and third $\widehat M_y$-odd bands is lifted, and the pair of NPs disappears. Consequently, the original crossed nodal rings split into two new isolated rings. For the upper nodal ring (orange), the two degenerate bands are significantly tilted in the direction ${\hat{\mathbf k}}_ \bot$ perpendicular to the ring such that both their perpendicular group velocities $v_ \bot ^{{\mathrm{even}}/{\mathrm{odd}}} = \nabla _{\mathbf{k}}\omega ^{{\mathrm{even}}/{\mathrm{odd}}} \cdot {\hat{\mathbf k}}_ \bot$ always have the same sign at any point on the ring, forming a type-II nodal ring^19–21^. As the lower $\widehat M_y$-odd band has a saddle-shaped dispersion, both type-I points (i.e., $v_ \bot ^{{\mathrm{odd}}}$ and $v_ \bot ^{{\mathrm{even}}}$ of opposite signs) and type-II points coexist on the lower nodal ring (red), and such band crossings are referred to as a type-III nodal ring^19^ or a hybrid nodal ring^20^.

### Spin-1 conical diffraction

It is well known that light beams travelling along the optical axes in biaxial crystals will undergo conical diffraction^45^. The conical diffraction phenomenon is actually a generic scattering effect for twofold degenerate Dirac points^46^ and should also occur for light scattered by almost any linearly crossing point on the nodal rings in our system. Specifically, when an incident light beam has frequency and momentum that match a certain point on the nodal rings, the refractive waves spread into a hollow cone in the PhC, and at the same time, the polarizations circling the cone trace out a great circle on the Poincaré sphere, manifesting the quantized *π* Berry phase encircling the nodal ring.

**Fig. 4:**
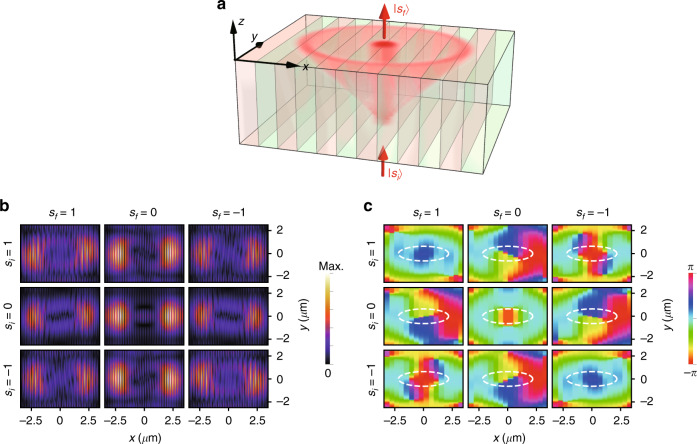
Spin-1 conical diffraction at a nexus point. **a** Schematic of spin-1 conical diffraction for a monochromatic beam with frequency *ω* = *ω*^NP^ incident on the PhC along the *z*-axis. The hollow cone illustrates the envelope of the trajectories corresponding to the wave components on the two conical iso-frequency surfaces around the NP. The straight light beam in the PhC corresponds to the wave components on the flat iso-frequency surface. Simulated **b** intensity and **c** phase (average of each period in the *x* direction) distribution of different output spin components (column indices) on the horizontal *xy* plane as the incident spin eigenstate of $\hat S_x$ (row indices) varies, where the parameters of the PhC are $\varepsilon _1 = \varepsilon _2 = 2,\varepsilon _3 = 1,\theta = \pi /6$ and *L* = 0.43 *μ*m. The vacuum wavelength of the incident beam is 0.713 *μ*m. The distance between the incident and output planes is 4 *μ*m

In contrast, the diffraction at the triple NPs appears strikingly different from that at other points on the NLs. Since the iso-frequency surfaces around each NP form a spin-1 Dirac-like cone (Fig. 3e), the monochromatic dynamics at an NP, e.g., ${\mathbf{k}}^{{\mathrm{NP}}_ + }$, is effectively described by a Schrödinger equation with the 2D spin-1 Hamiltonian $\hat H(\delta {\mathbf{k}}_{xy}) = v_x\hat S_z\delta k_x + \tilde v_y\hat S_y\delta k_y$ (here, $\tilde v_y = \sqrt {v_z^{{\mathrm{even}}}{\mathrm{/}}v_z^{{\mathrm{odd}}}} \,v_y$):

11 $$\begin{array}{*{20}{c}}\displaystyle {i\,v_z^{{\mathrm{odd}}}\frac{\partial }{{\partial z}}\left| \psi \right\rangle = \hat H\left( {\delta {\mathbf{k}}_{xy}} \right)\left| \psi \right\rangle } \end{array}$$

where the *z* coordinate serves as pseudo-time. Therefore, waves incident at the NPs should experience unconventional spin-1 conical diffraction^40^ rather than the spin-1/2-type diffraction at ordinary diabolic points. A schematic of spin-1 conical diffraction is shown in Fig. 4a, where a light beam with frequency *ω*^NP^ is incident along the *z*-axis in the PhC, and its wave vector spectrum concentrates near an NP. Since the NP is a singularity of group velocity for wave components on the two conical bands, these components will spread over a conical surface, whereas the components on the flat band will propagate straight along the *z*-axis. More interestingly, if the initial state of the beam is an eigenstate $\left| {s_i} \right\rangle$ of $\hat S_x$ with spin quantum number $s_i \in \{ - 1,0,1\}$, then the spin-1 character is inherent in the transition amplitude from $\left| {s_i} \right\rangle$ to another eigenstate $| {s_f} \rangle$ of $\hat S_x$ as the output of the diffraction process (see Supplementary Information S8):

12 $$\begin{array}{*{20}{c}} {\langle {s_f} |e^{ - i\hat H\left( {\delta {\mathbf{k}}_{xy}} \right)z/v_z^{{\mathrm{odd}}}}\left| {s_i} \right\rangle \propto \exp\left[ {i\left( {s_f - s_i} \right)\phi \left( {\delta {\mathbf{k}}_{xy}} \right)} \right]} \end{array}$$

where $\phi (\delta {\mathbf{k}}_{xy})$ denotes the polar angle of $(v_x\delta k_x + i\,\tilde v_y\delta k_y)$. Equation (12) shows that the phase of the output field winds *l* = *s*_*f*_ − *s*_*i*_ times around *δ***k**_*xy*_ = 0 in momentum space. Because the trajectories of the wave components encircling *δ***k**_*xy*_ = 0 also wrap around the *z*-axis in real space, the output field projected onto $| {s_f} \rangle$ will generate an optical vortex on the ring-shaped section of the diffractive cone, and the charge of the vortex, $l = s_f - s_i \in \{ 0, \pm 1, \pm 2\}$, is determined by the difference in the spin quantum number between the final and initial spin states^40^, which essentially reflects the conservation of the total generalized angular momentum for the spin-1 Hamiltonian (see Supplementary Information S8).

We have performed full-wave simulations for the nine possible combinations of input and output spin eigenstates. The intensities and phases of the output fields on a horizontal plane are shown in Fig. 4b, c, respectively, where the asymmetric intensity distributions originate from the anisotropy of the Dirac-like dispersion. In each panel of Fig. 4c, the white dashed ring corresponds to the section of the diffractive cone in the geometric optics approximation, and the winding number of the phase along the ring defines the charge of the optical vortex. We can see that the winding numbers agree with the theoretical predictions *l* = *s*_*f*_ − *s*_*i*_ in all panels, so we have demonstrated that spin-1 conical diffraction occurs in the PhC, which also indicates that the NPs can be used as a new route for studying 2D spin-1 dynamics.

## Discussion

We have discovered that a class of simple layer-stacked PhCs manifests a hidden symmetry of Maxwell’s equations, which directly influences the connectivity of photonic bands and engenders a pair of triply degenerate NPs where three symmetry-enforced NLs intersect. These photonic NPs not only are worthy of theoretical investigation as a novel kind of magnetic monopole that terminates Berry flux strings in momentum space but also induce exotic bulk transport effects in the PhC, which may lead to prospective applications. In particular, we found that the unusual spin-1 Dirac-like dispersion of the iso-frequency surfaces near an NP can induce spin-1 conical diffraction of optical beams, which can be used to generate optical vortices with a maximum topological charge of 2.

Our work takes the first step towards new research directions. On the one hand, the hidden symmetry of Maxwell’s equations reveals a novel mechanism for realizing protected degeneracies unique to photonic bands. The hidden symmetry here stems from the fractional periods of different components of ${\overleftrightarrow{\varepsilon}}_r$, which reflects a geometric property of the PhC but cannot be described by the conventional space groups. It would be fundamentally significant to develop a generalized space group theory including such symmetries for photonic systems. On the other hand, our proposed PhC consists of a single anisotropic dielectric material, but the band peculiarity comes entirely from the nontrivial periodic rotation of the optical axes inside the material. Although recent studies have shown that artificial gauge fields^47,48^, Pancharatnam-Berry phases^49^, and synthetic spin-orbit interactions^50^ for light can be achieved by arranging the dielectric polarization, there are still few works that study the photonic band topology of PhCs made of anisotropic dielectrics. Our results suggest that the intrinsic material anisotropy has irreplaceable features compared to the structural anisotropy, and anisotropic dielectrics, such as liquid crystals^49,50^, could become a platform for probing the unique topological effects of EM fields.

## Materials and methods

The methods for calculating the band structures include the transfer matrix approach for the PhC made of homogeneous anisotropic dielectric layers, with the results shown in Figs. 1 and 3 (see Supplementary Information S1 for details), and the plane wave expansion method for the PhC made of generic inhomogeneous dielectrics, with the results shown in Fig. 2. The numerical results of the spin-1 conical diffraction in Fig. 4 are simulated using the commercial software COMSOL Multiphysics. The input state with a certain spin quantum number is achieved by setting the field distribution on the lower boundary of the PhC as the superposition of the exact Bloch eigenstates according to the **k** · **p** Hamiltonian in Eq. (10). The output fields projected onto the spin eigenstates are averaged along the *x* direction in each period (a pair of AB layers), and the phase distributions shown in Fig. 4c correspond to these averaged fields.

## Supplementary information

Supplementary Information for Hidden-symmetry-enforced nexus points of nodal lines in layer-stacked dielectric photonic crystals

## Data Availability

The authors declare that all data supporting the findings of this study are available from the corresponding authors upon reasonable request.
