# Supplementary material for: Hidden-symmetry-enforced nexus points of nodal lines in layer-stacked dielectric photonic crystals
Source: Light Sci Appl. 2020 Oct 19;9:176. doi: 10.1038/s41377-020-00382-9 (PMC7572392; doi:10.1038/s41377-020-00382-9)
Supplement: Supplementary file 1 — Supplementary Information for Hidden-symmetry-enforced nexus points of nodal lines in layer-stacked dielectric photonic crystals [file 41377_2020_382_MOESM1_ESM.pdf]

# Supplementary information for "Hidden-symmetry-enforced nexus points of nodal lines in layer-stacked dielectric photonic crystals"

Zhongfei Xiong,[1,*] Ruo-Yang Zhang,[2,*] Rui Yu,[3] C. T. Chan,[2,†] and Yuntian Chen[1,4,‡]

[1] *School of Optical and Electronic Information, Huazhong University of Science and Technology, Wuhan 430074, China*

[2] *Department of Physics, The Hong Kong University of Science and Technology, Clear Water Bay, Hong Kong, China*

[3] *School of Physics and Technology, Wuhan University, Wuhan 430072, China*

[4] *Wuhan National Laboratory of Optoelectronics, Huazhong University of Science and Technology, Wuhan 430074, China*

## Contents

**S1.** Calculating Band structure using transfer matrix approach 2

**S2.** Hidden symmetries in the $k_y = 0$ plane 3

A. Hidden symmetries in the $\hat{M}_y$-odd subspace 4

B. Generalized fractional translation symmetry in the $\hat{M}_y$-even subspace 4

C. Generalized 1/4-period translation and 1/4-period twofold screw symmetries in the $k_y = 0$ plane 5

**S3.** Kramers degeneracies induced by the generalized 1/4-period twofold screw symmetry 6

**S4.** Determining the branch indices of the two bands connected to $|\mathbf{k}| = \omega = 0$ 8

**S5.** Asymptotic dispersion of bands at infinity 8

**S6.** Robustness of nexus points against the variation of materials 9

**S7.** Derivation of $\mathbf{k}\cdot\mathbf{p}$ Hamiltonian for layer-stacked photonic crystals 12

A. $\mathbf{k}\cdot\mathbf{p}$ Hamiltonian near the nexus points 12

**S8.** Spin-1 conical diffraction 13

* These authors contributed equally to this work.

† phchan@ust.hk

‡ yuntian@hust.edu.cn

## S1. Calculating Band structure using transfer matrix approach

In this section, we derive the analytical expressions of the band structures in the $k_y = 0$ plane for the AB-layer-stacked PhC. For a given angular frequency $\omega$ and a wavevector $\boldsymbol{\kappa} = (\kappa_x, 0, k_z)$ in the $k_y = 0$ plane, there are two plane wave eigensolutions in the homogeneous anisotropic medium described by Eq. (2) in the main text. The two plane wave solutions can be labeled by their $\hat{M}_y$ parities, and their dispersion relations are given by

$$\hat{M}_y-\text{odd}: \quad \kappa_x^2 + k_z^2 - \varepsilon_{yy}k_0^2 = 0 \qquad \Rightarrow \quad \kappa_{x\pm}^{\text{odd}} = \pm\kappa_1 = \pm\sqrt{\varepsilon_{yy}k_0^2 - k_z^2}, \tag{1}$$

$$\hat{M}_y-\text{even}: \quad \varepsilon_{xx}\kappa_x^2 + 2\varepsilon_{xz}\kappa_x k_z + \varepsilon_{zz}k_z^2 - \varepsilon_1\varepsilon_3 k_0^2 = 0 \quad \Rightarrow \quad \kappa_{x\pm}^{\text{even}} = \pm\kappa_2 - \frac{\varepsilon_{xz}}{\varepsilon_{xx}}k_z = \pm\frac{\sqrt{\varepsilon_1\varepsilon_3(\varepsilon_{xx}k_0^2 - k_z^2)}}{\varepsilon_{xx}} - \frac{\varepsilon_{xz}}{\varepsilon_{xx}}k_z, \tag{2}$$

where $k_0 = \omega/c$, $\varepsilon_1\varepsilon_3 \equiv \varepsilon_{xx}\varepsilon_{zz} - \varepsilon_{xz}^2$, and we note that $\varepsilon_{xz} = \pm g$ in layer A and layer B respectively. The corresponding eigenvectors are

$$\hat{M}_y-\text{odd}: \quad \phi_{\pm}^{\text{odd}} = (E_y, H_x, H_z)^\top = \left(\sqrt{\varepsilon_{yy}}k_0, -k_z, \pm\kappa_1\right)^\top \exp\left[i(\pm\kappa_1 x + k_z z)\right], \tag{3}$$

$$\hat{M}_y-\text{even}: \quad \phi_{\pm}^{\text{even}} = (E_x, E_z, H_y)^\top = \left(\pm\frac{\varepsilon_{xz}\kappa_2}{\varepsilon_1\varepsilon_3} + \frac{k_z}{\varepsilon_{xx}}, \mp\frac{\varepsilon_{xx}\kappa_2}{\varepsilon_1\varepsilon_3}, k_0\right)^\top \exp\left[i(\kappa_{x\pm}^{\text{even}} x + k_z z)\right]. \tag{4}$$

The Bloch eigenfunctions with certain $\hat{M}_y$ parity in the first period of the PhC ($x \in [-L/2, L/2]$) can be expressed as the superpositions of the plane wave fields in Eqs. (3) and (4):

$$\hat{M}_y-\text{odd}: \qquad \psi_\alpha^{\text{odd}} = a_\alpha\, \phi_{+,\alpha}^{\text{odd}} + b_\alpha\, \phi_{-,\alpha}^{\text{odd}}, \tag{5}$$

$$\hat{M}_y-\text{even}: \qquad \psi_\alpha^{\text{even}} = c_\alpha\, \phi_{+,\alpha}^{\text{even}} + d_\alpha\, \phi_{-,\alpha}^{\text{even}}. \tag{6}$$

where $\alpha = A, B$ labels the fields in layer A ($x \in [-L/2, 0]$) and layer B ($x \in [0, L/2]$) respectively. And according to the Bloch condition, the field in the $m^{\text{th}}$ period is given by $\psi_\alpha(x + mL) = \psi_\alpha(x)e^{imL\,k_x}$ with $k_x$ denoting the $x$ component of the Bloch wavevector inside the 1st Brillouin zone (BZ).

From the continuity conditions of $(E_y, H_z)$ and $(E_z, H_y)$ at the intracell interface $x = 0$, we obtain

$$\begin{pmatrix} 1 & 1 & 0 & 0 \\ 1 & -1 & 0 & 0 \\ 0 & 0 & -1 & 1 \\ 0 & 0 & 1 & 1 \end{pmatrix} \begin{pmatrix} a_A \\ b_A \\ c_A \\ d_A \end{pmatrix} = \begin{pmatrix} 1 & 1 & 0 & 0 \\ 1 & -1 & 0 & 0 \\ 0 & 0 & -1 & 1 \\ 0 & 0 & 1 & 1 \end{pmatrix} \begin{pmatrix} a_B \\ b_B \\ c_B \\ d_B \end{pmatrix}, \tag{7}$$

which shows that $(a_A, b_A, c_A, d_A)^\top = (a_B, b_B, c_B, d_B)^\top$. Similarly, from the continuity boundary conditions at the intercell interface $x = L/2$, we obtian

$$\begin{pmatrix} e^{i\kappa_1 L/2} & e^{-i\kappa_1 L/2} & 0 & 0 \\ e^{i\kappa_1 L/2} & -e^{-i\kappa_1 L/2} & 0 & 0 \\ 0 & 0 & -e^{i\tilde{\kappa}_+ L/2} & e^{-i\tilde{\kappa}_- L/2} \\ 0 & 0 & e^{i\tilde{\kappa}_+ L/2} & e^{-i\tilde{\kappa}_- L/2} \end{pmatrix} \begin{pmatrix} a_B \\ b_B \\ c_B \\ d_B \end{pmatrix} = e^{iL\,k_x} \begin{pmatrix} e^{-i\kappa_1 L/2} & e^{i\kappa_1 L/2} & 0 & 0 \\ e^{-i\kappa_1 L/2} & -e^{i\kappa_1 L/2} & 0 & 0 \\ 0 & 0 & -e^{-i\tilde{\kappa}_- L/2} & e^{i\tilde{\kappa}_+ L/2} \\ 0 & 0 & e^{-i\tilde{\kappa}_- L/2} & e^{i\tilde{\kappa}_+ L/2} \end{pmatrix} \begin{pmatrix} a_A \\ b_A \\ c_A \\ d_A \end{pmatrix}, \tag{8}$$

where $\tilde{k}_\pm = \kappa_2 \pm g k_z/\varepsilon_{xx}$. As a result, the transformer matrices for $\hat{M}_y$-odd and even modes can be written as

$$\hat{M}_y-\text{odd}: \qquad \begin{pmatrix} e^{i\kappa_1 L} & 0 \\ 0 & e^{-i\kappa_1 L} \end{pmatrix} \begin{pmatrix} a_A \\ b_A \end{pmatrix} = e^{iL\,k_x} \begin{pmatrix} a_A \\ b_A \end{pmatrix}, \tag{9}$$

$$\hat{M}_y-\text{even}: \qquad \begin{pmatrix} e^{i\kappa_2 L} & 0 \\ 0 & e^{-i\kappa_2 L} \end{pmatrix} \begin{pmatrix} c_A \\ d_A \end{pmatrix} = e^{iL\,k_x} \begin{pmatrix} c_A \\ d_A \end{pmatrix}. \tag{10}$$

Solving the two equations, we find that the Bloch wavevector in the $x$ direction takes the simple expression $k_x = (\pm\kappa_1 \bmod 2\pi)$ for odd modes and $k_x = (\pm\kappa_2 \bmod 2\pi)$ for even modes. Therefore, the dispersions of $\hat{M}_y$-odd and even bands in the $k_y = 0$ plane read

$$\hat{M}_y-\text{odd}: \qquad (\omega_m^{\text{odd}})^2/c^2 = \frac{1}{\varepsilon_{yy}}\left[\left(k_x + m\frac{2\pi}{L}\right)^2 + k_z^2\right], \tag{11}$$

$$\hat{M}_y-\text{even}: \qquad (\omega_m^{\text{even}})^2/c^2 = \frac{\varepsilon_{xx}}{\varepsilon_1\varepsilon_3}\left(k_x + m\frac{2\pi}{L}\right)^2 + \frac{1}{\varepsilon_{xx}}k_z^2, \tag{12}$$

with $m \in \mathbb{Z}$ numbering the bands. And the corresponding normalized Bloch states are

$$\hat{M}_y - \text{odd}: \quad \psi_m^{\text{odd}} = \left(E_y, H_z, H_z\right)^\top = \frac{1}{\sqrt{2\varepsilon_{yy}L}}\left(1, -\frac{ck_z}{\omega_m^{\text{odd}}}, \frac{c(k_x + m\frac{2\pi}{L})}{\omega_m^{\text{odd}}}\right)^\top \exp\left[i\left(k_x + m\frac{2\pi}{L}\right)x + k_z z\right)\right], \tag{13}$$

$$\begin{aligned}\hat{M}_y - \text{even}: \quad \psi_m^{even} = \left(E_x, E_z, H_y\right)^\top = \frac{c}{\sqrt{2L}\,\omega_m^{\text{even}}}&\left(\frac{\varepsilon_{xz}(k_x + m\frac{2\pi}{L})}{\varepsilon_1\varepsilon_3} + \frac{k_z}{\varepsilon_{xx}}, -\frac{\varepsilon_{xx}(k_x + m\frac{2\pi}{L})}{\varepsilon_1\varepsilon_3}, \frac{\omega_m^{\text{even}}}{c}\right)^\top \\ &\cdot \exp\left[i\left((k_x + n\frac{2\pi}{L}) - \frac{\varepsilon_{xz}}{\varepsilon_{xx}}k_z\right)x + ik_z z\right],\end{aligned} \tag{14}$$

Eqs. (11) and (12) exhibit that all $\hat{M}_y$-odd (even) bands have identical conical dispersions up to a translation along the $x$-axis in the extended BZ. Therefore, any pair of bands with opposite mirror parities will cross each other along a nodal ling in the $k_y = 0$ plane. We note that the nodal line can be any kinds of conic sections, including ellipse, parabola, and hyperbola, depending on the parameters of the PhC. In particular, the two red nodal rings of our interest in the main text correspond to the intersections of the even band of $m^{\text{even}} = 0$ and the odd bands of $m^{\text{odd}} = \pm 1$, given by the following implicit equation:

$$\frac{\varepsilon_{xx} - \varepsilon_{yy}}{\varepsilon_{xx}\varepsilon_{yy}}k_z^2 = \frac{\varepsilon_{xx}}{\varepsilon_1\varepsilon_3}k_x^2 - \frac{1}{\varepsilon_{yy}}\left(k_x^2 \pm \frac{2\pi}{L}\right)^2. \tag{15}$$

And from the Eqs. (11) and (12) , we can also obtain the frequency and wavevectors of the pair of triply degenerate crossing points of the two nodal rings, *i.e.* the triply degenerate nexus points: $\omega^{\text{NP}} = \frac{2\pi c}{L\sqrt{\varepsilon_{yy} - \varepsilon_{xx}}}$ and $\mathbf{k}^{\text{NP}_\pm} = (0, 0, \pm\frac{2\pi}{L}\sqrt{\frac{\varepsilon_{xx}}{\varepsilon_{yy} - \varepsilon_{xx}}})$.

## S2. Hidden symmetries in the $k_y = 0$ plane

In this section, we discuss the hidden symmetry in the $k_y = 0$ plane induced by the fractional periodicity of the components of constitutive tensors. In this subsystem, the Maxwell's equations in the layer-stacked dielectric PhC can be written as

$$\underbrace{\left(\begin{array}{c|c} 0 & \begin{matrix} 0 & k_z & 0 \\ -k_z & 0 & -i\partial_x \\ 0 & i\partial_x & 0 \end{matrix} \\ \hline \begin{matrix} 0 & -k_z & 0 \\ k_z & 0 & i\partial_x \\ 0 & -i\partial_x & 0 \end{matrix} & 0 \end{array}\right)}_{\hat{\mathcal{N}}} \underbrace{\begin{pmatrix} \mathbf{E} \\ \mathbf{H} \end{pmatrix}}_{\Psi} = \omega \underbrace{\begin{pmatrix} \varepsilon_0 \overleftrightarrow{\varepsilon}_r(x) & 0 \\ 0 & \mu_0 \hat{I}_{3\times 3} \end{pmatrix}}_{\hat{\mathcal{M}}(x)} \begin{pmatrix} \mathbf{E} \\ \mathbf{H} \end{pmatrix}, \tag{16}$$

where $\overleftrightarrow{\varepsilon}_r$ takes the form of Eq. (2) in the main text. And hereinafter, we adopt the natural units with $\varepsilon_0 = \mu_0 = 1$ for convenience. In general, the Maxwell's equations are invariant under a symmetry transformation $\widetilde{A}$, as long as

$$\widetilde{A}\hat{\mathcal{N}}\widetilde{A}^{-1} = \hat{C}\hat{\mathcal{N}} \quad \text{and} \quad \widetilde{A}\hat{\mathcal{M}}\widetilde{A}^{-1} = \hat{C}\hat{\mathcal{M}}, \tag{17}$$

where $\hat{C}$ can be an arbitrary invertible operator. However, for space group symmetries of the structure, $\hat{C}$ is fixed as identity. If the constitutive tensor $\hat{\mathcal{M}}$ is invertible, which is always true for dielectric PhCs, Eq. (17) is equivalent to the invariance of the effective Hamiltonian $\hat{H} = \hat{\mathcal{M}}^{-1}\hat{\mathcal{N}}$, *i.e.* $\widetilde{A}\hat{H}\widetilde{A}^{-1} = \hat{H}$. In the $k_y = 0$ plane, the effective Hamiltonian reads

$$\hat{H}(k_y = 0) = \hat{\mathcal{M}}^{-1}\hat{\mathcal{N}} = \left(\begin{array}{c|c} 0 & \hat{H}_1 \\ \hline \hat{H}_2 & 0 \end{array}\right) = \left(\begin{array}{c|c} 0 & \begin{matrix} 0 & \frac{\varepsilon_{xz}}{\varepsilon_1\varepsilon_3}(-i\partial_x) + \frac{\varepsilon_{zz}}{\varepsilon_1\varepsilon_3}k_z & 0 \\ -\frac{k_z}{\varepsilon_{yy}} & 0 & \frac{1}{\varepsilon_{yy}}(-i\partial_x) \\ 0 & \frac{\varepsilon_{xx}}{\varepsilon_1\varepsilon_3}(i\partial_x) - \frac{\varepsilon_{xz}}{\varepsilon_1\varepsilon_3}k_z & 0 \end{matrix} \\ \hline \begin{matrix} 0 & -k_z & 0 \\ k_z & 0 & i\partial_x \\ 0 & -i\partial_x & 0 \end{matrix} & 0 \end{array}\right). \tag{18}$$

In what follows, we show that the fractional periodicity of the elements of $\overleftrightarrow{\varepsilon}_r$ can give rise to a hidden symmetry of $\hat{H}(k_y = 0)$ beyond space groups.

### A. Hidden symmetries in the $\hat{M}_y$-odd subspace

In the $\hat{M}_y$-odd subspace, since the electric field is polarized in the $y$ direction, the $\hat{M}_y$-odd band structure is entirely determined by $\varepsilon_{yy}$. Especially, for the AB-layered PhC in Fig. 1 of the main text, $\varepsilon_{yy}$ is a global constant, and hence the band structure of the odd modes on the $k_y = 0$ plane is directly obtained by folding the light cone in a homogeneous medium with a constant permittivity. Consequently, all $\hat{M}_y$-odd bands are twofold degenerate along $\Gamma - Z$ except for the one connected to $\omega = |\mathbf{k}| = 0$ point.

In fact, we can relax the condition of a constant $\varepsilon_{yy}$ so that the period of $\varepsilon_{yy}$ is a fraction $1/N$ of the primitive period $L$ of the whole PhC, namely $\varepsilon_{yy}(x + L/N) = \varepsilon_{yy}(x)$, the width of the genuine BZ of the $\hat{M}_y$-odd subspace (marked as BZ(odd)) should be $2N\pi/L$ which is $N$ times as large as the primitive BZ of the whole system (marked as BZ(whole)). Therefore, the finial band structure in BZ(whole) is obtained by translating the bands in BZ(odd) periodically with spacing $\Delta x = 2\pi/L$. As a result, twofold degenerate Kramers-like NLs can appear along $\Gamma - Z$ as long as $N \geq 3$. If we also require the PhC respects the space group $\mathbb{R}^2 \rtimes \mathrm{Rod}(22)$, $N$ should be an even number and thus the minimal value of $N$ is 4. We introduce the 1/4-period translation operator in the $\hat{M}_y$-odd subspace:

$$\hat{T}_x^{\mathrm{odd}}(L/4) = \hat{T}_x(L/4)\hat{P}_- + \hat{P}_+, \tag{19}$$

where $\hat{T}_x(L/4)$ denotes the original translation operator, and $\hat{P}_\pm = \frac{1}{2}(\hat{I} \pm \hat{M}_y)$ is the projection operator onto $\hat{M}_y$-even(+)/odd(-) subspace. So the fractional periodicity of $\varepsilon_{yy}$ is equivalent to $\hat{T}_x^{\mathrm{odd}}(L/4)\hat{H}(k_y = 0)\hat{T}_x^{\mathrm{odd}}(L/4)^{-1} = \hat{H}(k_y = 0)$. At the same time, the $\hat{M}_y$-odd subsapce is also invariant under a generalized 1/4-period twofold screw operation about the $x$ axis:

$$\widetilde{S}_{L/4}^{\mathrm{odd}} = \left(\hat{C}_{2x}\hat{T}_x(L/4)\right)\hat{P}_- + \hat{P}_+, \tag{20}$$

namely, the combination of a 1/4-period translation and a twofold rotation about the $x$ axis acting on the odd subspace. We will prove in the next section that the generalized 1/4-period twofold screw symmetry, together with the time reversal symmetry, guarantees the emergence of the Kramers-like NLs along $\Gamma - Z$.

### B. Generalized fractional translation symmetry in the $\hat{M}_y$-even subspace

For the $\hat{M}_y$-even subspace, it is convenient to deal with the sub-Hamiltonian of Eq. (18) acting on $\psi^{\mathrm{even}} = (E_x, E_z, H_y)^\intercal$:

$$\hat{H}_{\mathrm{even}} = \begin{pmatrix} 0 & 0 & \frac{\varepsilon_{xz}}{\varepsilon_1\varepsilon_3}(-i\partial_x) + \frac{\varepsilon_{zz}}{\varepsilon_1\varepsilon_3}k_z \\ 0 & 0 & \frac{\varepsilon_{xx}}{\varepsilon_1\varepsilon_3}(i\partial_x) - \frac{\varepsilon_{xz}}{\varepsilon_1\varepsilon_3}k_z \\ k_z & i\partial_x & 0 \end{pmatrix}. \tag{21}$$

Though the period of the sub-Hamiltonian is the same as the full system, we will show that it can be converted into a new form with fractional period via a similarity transformation. First, we change the eigen basis from $\psi^{\mathrm{even}} = (E_x, E_z, H_y)^\intercal$ to $\psi'^{\mathrm{even}} = \hat{G}(x)\psi^{\mathrm{even}} = (D_x, E_z, H_y)^\intercal$ with the transformation matrix:

$$\hat{G}(x) = \begin{pmatrix} \varepsilon_{xx}(x) & \varepsilon_{xz}(x) & 0 \\ 0 & 1 & 0 \\ 0 & 0 & 1 \end{pmatrix}, \tag{22}$$

and the sub-Hamiltonian is transformed accordingly:

$$\hat{H}'_{\mathrm{even}} = \hat{G}\hat{H}_{\mathrm{even}}\hat{G}^{-1} = \begin{pmatrix} 0 & 0 & k_z \\ 0 & 0 & -\frac{\varepsilon_{xx}}{\varepsilon_1\varepsilon_3}\left((-i\partial_x) + \frac{\varepsilon_{xz}}{\varepsilon_{xx}}k_z\right) \\ \frac{k_z}{\varepsilon_{xx}} & -\left((-i\partial_x) + \frac{\varepsilon_{xz}}{\varepsilon_{xx}}k_z\right) & 0 \end{pmatrix}. \tag{23}$$

It shows that $-i\partial_x$ always appears together with an effective gauge potential $\mathcal{A}_x = k_z\frac{\varepsilon_{xz}}{\varepsilon_{xx}}$ in $\hat{H}'_{\mathrm{even}}$. Therefore, we can use a $U(1)$ gauge transformation $\hat{U}(x, k_z) = \exp\left[i\int_0^x \mathcal{A}_x(\xi)d\xi\right] = \exp\left[ik_z\int_0^x \frac{\varepsilon_{xz}(\xi)}{\varepsilon_{xx}(\xi)}d\xi\right]$ to remove the effective gauge

potential in the Hamiltonian:

$$\hat{H}''_{\text{even}} = \hat{U}\hat{H}'_{\text{even}}\hat{U}^\dagger = \hat{U}\hat{G}\hat{H}_{\text{even}}\hat{G}^{-1}\hat{U}^\dagger = \begin{pmatrix} 0 & 0 & k_z \\ 0 & 0 & \frac{\varepsilon_{xx}}{\varepsilon_1\varepsilon_3}(i\partial_x) \\ \frac{k_z}{\varepsilon_{xx}} & i\partial_x & 0 \end{pmatrix}. \tag{24}$$

We note that the gauge transformation is $k_z$ dependent. Under the coordinate $z$ representation, it appears as a translation operator with $x$-dependent translation along the $z$ axis:

$$\hat{U}(x) = \int dk_z|k_z\rangle\langle k_z| \exp\left[ik_z \int_0^x \frac{\varepsilon_{xz}(\xi)}{\varepsilon_{xx}(\xi)}d\xi\right] = \exp\left[\left(\int_0^x \frac{\varepsilon_{xz}(\xi)}{\varepsilon_{xx}(\xi)}d\xi\right)\partial_z\right] = \hat{T}\left(-\left(\int_0^x \frac{\varepsilon_{xz}(\xi)}{\varepsilon_{xx}(\xi)}d\xi\right)\mathbf{e}_z\right). \tag{25}$$

After the combined similarity transformation $(\hat{U}\hat{G})$, $\hat{H}''_{\text{even}}$ is only explicitly dependent on $\varepsilon_{xx}$ and $\varepsilon_1\varepsilon_3 = \varepsilon_{xx}\varepsilon_{zz} - (\varepsilon_{xz})^2$ as shown in Eq. (24). For the AB-layer-stacked PhC shown in Fig. 1 of the main text, both $\varepsilon_{xx}$ and $\varepsilon_1\varepsilon_3$ are constant. Therefore, the $\hat{M}_y$-even band structure are also obtained by folding the light cone of a homogeneous system, and all bands are twofold generate along $\Gamma - Z$ except for the lowest one attached at $\omega = |\mathbf{k}| = 0$.

Similar to the $\hat{M}_y$-odd case, if we relax the condition to that the common period of $\varepsilon_{xx}(x)$ and $\varepsilon_1(x)\varepsilon_3(x)$ is a fraction $1/N$ of the full period $L$ of the PhC with $N \geq 3$, twofold degeneracies of even bands can still appear along the $\Gamma - Z$ line. And without changing the space group of the system, the minimal value of $N$ is 4 and the corresponding components of the permittivity should satisfy

$$\varepsilon_{ii}(x + L/4) = \varepsilon_{ii}(x),\ (i = x, y, z), \quad \text{and} \quad \varepsilon_{xz}(x + L/4)^2 = \varepsilon_{xz}(x)^2,\ \varepsilon_{xz}(x + L/2) = -\varepsilon_{xz}(x). \tag{26}$$

In the following, we will focus on this special case with minimized constraints on the PhC that are compatible with the space group $\mathbb{R}^2 \rtimes \text{Rod}(22)$ and support the Kramers-like NLs along $\Gamma - Z$.

The $L/4$ periodicity of $\hat{H}''_{\text{even}}$ can be regarded as a generalized fractional translation symmetry operating on the $\hat{M}_y$-even subspace for the original Hamiltonian (18), $\widetilde{T}_x^{\text{even}}(L/4)\hat{H}(k_y = 0)\widetilde{T}_x^{\text{even}}(L/4)^{-1} = \hat{H}(k_y = 0)$, with

$$\widetilde{T}_x^{\text{even}}(L/4) = \left(\hat{G}^{-1}\hat{U}^\dagger\hat{T}_x(L/4)\hat{U}\hat{G}\right)\hat{P}_+ + \hat{P}_-, \tag{27}$$

where $\hat{G}$ is reformulated as $\hat{G} = \hat{I}_{6\times6} + (\varepsilon_{xx}(x) - 1)\hat{\mathbf{e}}_1\hat{\mathbf{e}}_1 + \varepsilon_{xz}(x)\hat{\mathbf{e}}_1\hat{\mathbf{e}}_3$ for the 6-dimensional eigenvector $\Psi = (\mathbf{E}, \mathbf{H})^\intercal$. Moreover, since $\hat{H}''_{\text{even}}$ also respects the twofold rotation symmetry $\hat{C}_{2x}$ about the $x$ axis, we can further introduce the generalized 1/4-period twofold screw operator:

$$\widetilde{S}^{\text{even}}_{L/4} = \left(\hat{G}^{-1}\hat{U}^\dagger\left(\hat{C}_{2x}\hat{T}_x(L/4)\right)\hat{U}\hat{G}\right)\hat{P}_+ + \hat{P}_-, \tag{28}$$

and the Hamiltonian (18) is invariant under $\hat{S}^{\text{even}}_{L/4}$.

### C. Generalized 1/4-period translation and 1/4-period twofold screw symmetries in the $k_y = 0$ plane

Combining Eqs. (19),(27) and Eqs. (20),(28) respectively, we obtain the generalized 1/4-period translation operator in the whole $k_y = 0$ plane:

$$\begin{aligned}\widetilde{T}_x(L/4) &= \widetilde{T}_x^{\text{even}}(L/4)\widetilde{T}_x^{\text{odd}}(L/4) = \hat{T}_x(L/4)\hat{P}_- + \left(\hat{G}^{-1}\hat{U}^\dagger\hat{T}_x(L/N)\hat{U}\hat{G}\right)\hat{P}_+ \\ &= \widetilde{U}^{-1}\hat{T}_x(L/4)\widetilde{U} = \left(\hat{P}_- + \hat{G}^{-1}\hat{U}^\dagger\hat{P}_+\right)\hat{T}_x(L/4)\left(\hat{P}_- + \hat{U}\hat{G}\hat{P}_+\right),\end{aligned} \tag{29}$$

and the generalized 1/4-period twofold screw operator in the $k_y = 0$ plane:

$$\widetilde{S}_{L/4} = \widetilde{S}^{\text{even}}_{L/4}\widetilde{S}^{\text{odd}}_{L/4} = \left(\hat{C}_{2x}\hat{T}_x(L/4)\right)\hat{P}_- + \left(\hat{G}^{-1}\hat{U}^\dagger\left(\hat{C}_{2x}\hat{T}_x(L/4)\right)\hat{U}\hat{G}\right)\hat{P}_+ = \widetilde{U}^{-1}\left(\hat{C}_{2x}\hat{T}_x(L/4)\right)\widetilde{U}, \tag{30}$$

where

$$\widetilde{U}(x,k_z) = \hat{P}_- + \hat{U}\hat{G}\hat{P}_+ = \left(\begin{array}{ccc|ccc} e^{i\varphi(x,k_z)}\varepsilon_{xx}(x) & 0 & e^{i\varphi(x,k_z)}\varepsilon_{xz}(x) & & & \\ 0 & 1 & 0 & & 0 & \\ 0 & 0 & e^{i\varphi(x,k_z)} & & & \\ \hline & & & 1 & 0 & 0 \\ & 0 & & 0 & e^{i\varphi(x,k_z)} & 0 \\ & & & 0 & 0 & 1 \end{array}\right) \tag{31}$$

with $\varphi(x,k_z) = k_z \int_0^x \frac{\varepsilon_{xz}(\xi)}{\varepsilon_{xx}(\xi)} d\xi$, and $\widetilde{U}^{-1} = \hat{P}_- + \hat{G}^{-1}\hat{U}^\dagger\hat{P}_+$. It can be directly checked that the effective Hamiltonian given in Eq. (18) is invariant under $\widetilde{T}_x(L/4)$ and $\widetilde{S}_{L/4}$, providing that the permittivity of the PhC satisfies Eq. (26),

$$\widetilde{T}_x(L/4)\hat{H}(k_y=0)\widetilde{T}_x(L/4)^{-1} = \hat{H}(k_y=0), \tag{32}$$

$$\widetilde{S}_{L/4}\hat{H}(k_y=0)\widetilde{S}_{L/4}^{-1} = \hat{H}(k_y=0). \tag{33}$$

Consequently, we have demonstrated that the fractional periodicity of the elements of permittivity tensor engenders the hidden symmetries of the Maxwell's equations. And we have the relation between the generalized 1/4-period screw rotation and the generalized fractional translation: $\widetilde{S}_{L/4}^2 = \widetilde{T}_x(L/4)^2 = \widetilde{T}_x(L/2)$. The generalized 1/4-period translation operator satisfies

$$\widetilde{T}_x(L/4)^\dagger = \widetilde{U}^\dagger\hat{T}_x(-L/4)(\widetilde{U}^{-1})^\dagger = \widetilde{U}^\dagger\widetilde{U}\left(\widetilde{U}^{-1}\hat{T}_x(-L/4)\widetilde{U}\right)\widetilde{U}^{-1}(\widetilde{U}^{-1})^\dagger = \left(\widetilde{U}^\dagger\widetilde{U}\right)\widetilde{T}_x(L/4)^{-1}\left(\widetilde{U}^\dagger\widetilde{U}\right)^{-1}, \tag{34}$$

and the similar result is established for the generalized screw rotation:

$$\widetilde{S}_{L/4}^\dagger = \left(\widetilde{U}^\dagger\widetilde{U}\right)\widetilde{S}_{L/4}^{-1}\left(\widetilde{U}^\dagger\widetilde{U}\right)^{-1}. \tag{35}$$

Since $\widetilde{U}^\dagger\widetilde{U}$ is hermitian and positive definite, both the two hidden symmetry operators are **($\widetilde{U}^\dagger\widetilde{U}$)-pseudo-unitary** and have unimodular eigenvalues [1]. In particular, since $\left(\widetilde{S}_{L/4}\right)^4 = \hat{T}_x(L)$, the eigenvalues of $\widetilde{S}_{L/4}$ for a Bloch state $\Psi(k_x,0,0)$ on the $k_x$-axis must be a forth root of $e^{ik_xL}$, *i.e.*

$$\widetilde{S}_{L/4}\Psi^{(s)}(k_x,0,0) = s\, e^{ik_xL/4}\,\Psi^{(s)}(k_x,0,0), \tag{36}$$

with $s = \pm 1, \pm i$ denoting the $\widetilde{S}_{L/4}$ **branch index** of the Bloch state. We note that the $\widetilde{S}_{L/4}$ branch index is only well defined for the states on the $k_x$-axis. Nevertheless, since $\widetilde{S}_{L/4}^2 = \widetilde{T}_x(L/2)$, we have

$$\widetilde{S}_{L/4}^2\Psi(k_x,0,k_z) = \widetilde{T}_x(L/2)\Psi(k_x,0,k_y) = \pm e^{ik_xL/2}\Psi(k_x,0,k_z), \tag{37}$$

$$\widetilde{S}_{L/4}^2\Psi^{(s)}(k_x,0,0) = s^2 e^{ik_xL/2}\Psi^{(s)}(k_x,0,0). \tag{38}$$

Therefore, the $\widetilde{S}_{L/4}^2$-parity (equal to the square of the branch index $s^2 = \pm 1$) is well defined for the whole band on the $k_y = 0$ plane, and is determined by the branch index of the states $\Psi^{(s)}(k_x,0,0)$ on that band. For convenience, we will assign a "**pseudo branch index**" for every Bloch state on the $k_y = 0$ plane as $\Psi^{(s)}(k_x,0,k_z)$, while we remind that only its square $s^2$ denoting the $\widetilde{S}_{L/4}^2$-parity of the state is meaningful in general (the sign of the pseudo branch index for a state with $k_z \neq 0$ is indeterminate, as $s^2 = (-s)^2$), but the sign of $s$ makes practical sense for the states on the $k_x$-axis.

### S3. Kramers degeneracies induced by the generalized 1/4-period twofold screw symmetry

Here, we express the time reversal operator in the coordinate-momentum mixed representation $(x,k_y,k_z)$: $\mathcal{T} = \hat{\tau}\mathcal{K}(k_y \to -k_y, k_z \to -k_z)$, where $\hat{\tau} = \text{diag}(\hat{I}_{3\times3}, -\hat{I}_{3\times3})$ and $\mathcal{K}$ denotes complex conjugate. It is easy to check that $\mathcal{T}$ commutes with $\widetilde{U}$ and $\widetilde{S}_{L/4}$: $[\mathcal{T},\widetilde{U}] = 0$, $[\mathcal{T},\widetilde{S}_{L/4}] = 0$. As introduced in the main text, the combination of the generalized 1/4-period screw rotation operator $\widetilde{S}_{L/4}$ and the time reversal gives a **($\widetilde{U}^\dagger\widetilde{U}$)-pseudo-antiunitary** symmetry operation for the layer-stacked PhC:

$$\hat{\Theta}_{L/4} = \mathcal{T}\widetilde{S}_{L/4}, \tag{39}$$

Here, $(\widetilde{U}^\dagger\widetilde{U})$-pseudo-antiunitarity means that

$$\langle\hat{\Theta}_{L/4}\psi|\widetilde{U}^\dagger\widetilde{U}|\hat{\Theta}_{L/4}\phi\rangle = \langle\psi|\widetilde{U}^\dagger\widetilde{U}|\phi\rangle^*, \tag{40}$$

where $\psi$, $\phi$ represent two arbitrary states.

$\hat{\Theta}_{L/4}$ operating on a Bloch state $\Psi^{(s)}(k_x,0,k_z)$ on $k_y=0$ plane yields a new Bloch state $\widetilde{\Psi}(-k_x,0,k_z)=\hat{\Theta}_{L/4}\Psi^{(s)}(k_x,0,k_z)$ of the same frequency at $(-k_x,0,k_z)$. In addition, since

$$\begin{aligned}\widetilde{S}_{L/4}\widetilde{\Psi}(-k_x,0,0) &= \widetilde{S}_{L/4}\hat{\Theta}_{L/4}\Psi^{(s)}(k_x,0,0) = \hat{\Theta}_{L/4}\widetilde{S}_{L/4}\Psi^{(s)}(k_x,0,0)\\ &= \widetilde{S}_{L/4}\left(se^{ik_xL/4}\Psi^{(s)}(k_x,0,0)\right) = s^*e^{-ik_xL/4}\widetilde{\Psi}(-k_x,0,0),\end{aligned} \tag{41}$$

the new Bloch state $\widetilde{\Psi}(-k_x,0,k_z)=\widetilde{\Psi}^{(s^*)}(-k_x,0,k_z)$ has pseudo branch index $s^*$ and has the same $\widetilde{S}^2_{L/4}$-parity $(s^*)^2=s^2$ as $\Psi^{(s)}(k_x,0,k_z)$.

In the mean time,

$$\hat{\Theta}^2_{L/4}\Psi^{(s)}(k_x,0,k_z) = \widetilde{S}^2_{L/4}\Psi^{(s)}(k_x,0,k_z) = s^2e^{ik_xL/2}\Psi^{(s)}(k_x,0,k_z). \tag{42}$$

In particular, on the $\Gamma-Z$ line ($k_x=k_y=0$), $\hat{\Theta}^2_{L/4}\Psi^{(s)}(0,0,k_z)=s^2\Psi^{(s)}(0,0,k_z)$. Consequently, if the $\widetilde{S}^2_{L/4}$-parity of the state equals $s^2=-1$ (*i.e.* pseudo branch index of $\Psi^{(s)}(0,k_z)$ is $s=\pm i$), we also have

$$\hat{\Theta}^2_{L/4}\Psi^{(\pm i)}(0,0,k_z) = -\Psi^{(\pm i)}(0,0,k_z), \tag{43}$$

so the pseudo-antiunitary operator $\hat{\Theta}_{L/4}$ serves as a pseudo-Fermionic time reversal symmetry for the Bloch states with $\widetilde{S}^2_{L/4}$-parity $s^2=-1$ on the $\Gamma-Z$ line. And according to the similar derivation of Kramers theorem, we have

$$\begin{aligned}\langle\widetilde{\Psi}^{(\mp i)}(0,0,k_z)|\widetilde{U}^\dagger\widetilde{U}|\Psi^{(\pm i)}(0,0,k_z)\rangle &= \langle\hat{\Theta}_{L/4}\Psi^{(\pm i)}|\widetilde{U}^\dagger\widetilde{U}|\Psi^{(\pm i)}\rangle = \langle\hat{\Theta}^2_{L/4}\Psi^{(\pm i)}|\widetilde{U}^\dagger\widetilde{U}|\hat{\Theta}_{L/4}\Psi^{(\pm i)}\rangle^*\\ &= -\langle\Psi^{(\pm i)}|\widetilde{U}^\dagger\widetilde{U}|\hat{\Theta}_{L/4}\Psi^{(\pm i)}\rangle^* = -\langle\Psi^{(\pm i)}|\widetilde{U}^\dagger\widetilde{U}|\hat{\Theta}_{L/4}\Psi^{(\pm i)}\rangle^\dagger\\ &= -\langle\widetilde{\Psi}^{(\mp i)}(0,0,k_z)|\widetilde{U}^\dagger\widetilde{U}|\Psi^{(\pm i)}(0,0,k_z)\rangle\\ \Rightarrow\qquad \langle\widetilde{\Psi}^{(\mp i)}(0,0,k_z)&|\widetilde{U}^\dagger\widetilde{U}|\Psi^{(\pm i)}(0,0,k_z)\rangle = 0,\end{aligned} \tag{44}$$

where the second equality of the first line is due to the pseudo-antiunitarity of $\hat{\Theta}_{L/4}$. Since $\widetilde{U}^\dagger\widetilde{U}$ is positive definite, $\langle\widetilde{\Psi}(0,k_z)^{(\mp i)}|\widetilde{U}^\dagger\widetilde{U}|\Psi^{(\pm i)}(0,0,k_z)\rangle=0$ indicates that $\widetilde{\Psi}^{(\mp i)}(0,0,k_z)=\hat{\Theta}_{L/4}\Psi^{(\pm i)}(0,0,k_z)$ **and** $\Psi^{(\pm i)}(0,0,k_z)$ **must be two distinct and degenerate Bloch states**. As a result, we have proved that **a Bloch band with negative** $\widetilde{S}^2_{L/4}$ **parity must intersect with another band with negative** $\widetilde{S}^2_{L/4}$ **parity along** $\Gamma-Z$**, forming Kramers-like NLs. Moreover, the pair of intersecting bands have certain branch indices** $s=i$ **and** $s=-i$ **along** $\Gamma-X$.

On the other hand, it is well known that the ordinary twofold screw symmetry $\hat{S}_{2x}$ together with the time reversal symmetry $\mathcal{T}$ protects all Bloch states on the $k_x=\pi/L$ plane are doubly degenerate. And the two degenerate states $\Psi(\pi/L,k_y,k_z)$ and $\widetilde{\Psi}(\pi/L,k_y,k_z)$ are correlated by the combined antiunitary operator $\hat{\Theta}_{L/2}=\mathcal{T}\hat{S}_{2x}$:

$$\widetilde{\Psi}(\pi/L,k_y,k_z) = \hat{\Theta}_{L/2}\Psi(\pi/L,k_y,k_z). \tag{45}$$

In particular, at X point ($\mathbf{k}=(\pi/L,0,0)$), if $\Psi^{(s)}(\pi/L,0,0)$ has branch index $s$, we have

$$\begin{aligned}\widetilde{S}_{L/4}\widetilde{\Psi}(\pi/L,0,0) &= \widetilde{S}_{L/4}\hat{\Theta}_{L/2}\Psi^{(s)}(\pi/L,0,0) = \hat{\Theta}_{L/2}\widetilde{S}_{L/4}\Psi^{(s)}(\pi/L,0,0)\\ &= \hat{\Theta}_{L/2}\left(se^{i\pi/4}\Psi^{(s)}(\pi/L,0,0)\right) = s^*e^{-i\pi/4}\hat{\Theta}_{L/2}\Psi^{(s)}(\pi/L,0,0)\\ &= (-is^*)e^{i\pi/4}\widetilde{\Psi}(\pi/L,0,0),\end{aligned} \tag{46}$$

where the second equality of the first line is due to the fact that $\widetilde{S}_{L/4}$ and $\hat{\Theta}_{L/2}$ are commutable. This result shows that $\widetilde{\Psi}(\pi/L,0,0)=\widetilde{\Psi}^{(-is^*)}(\pi/L,0,0)$ has branch index $-is^*$. In other words, **the pair of Bloch bands (along the** $k_x$**-axis) degenerate at** $X$ **must have branch indices of either** $s=1,s=-i$ **or** $s=-1,s=i$.

### S4. Determining the branch indices of the two bands connected to $|\mathbf{k}| = \omega = 0$

In this section, we figure out the branch indices of the two bands attached at $|\mathbf{k}| = \omega = 0$. The expansion of the Bloch states $\Psi(k_x, 0, 0)$ at $k_x = \omega = 0$ reads

$$\Psi(k_x, 0, 0) = e^{ik_x x} u_{k_x}(x) = e^{ik_x x} \left[ u_0(x) + \left. \frac{\partial u_{k_x}}{\partial k_x} \right|_{k_x=0} k_x + \mathcal{O}\left(k_x^2\right) \right], \tag{47}$$

with $u_0 = (\mathbf{e}^0, \mathbf{h}^0)^\top$. Substitution of the expansion into the Maxwell's equation (16) yields

$$\underbrace{\hat{\mathcal{N}}(-i\partial_x) u_0}_{\text{0-order: } \mathcal{O}(1)} + \underbrace{\left[ \hat{\mathcal{N}}(k_x) u_0 - \omega \hat{\mathcal{M}} u_0 + \hat{\mathcal{N}}(-i\partial_x) \left. \frac{\partial u_{k_x}}{\partial k_x} \right|_{k_x=0} k_x \right]}_{\text{1-order: } \mathcal{O}(k_x)} + \mathcal{O}\left(k_x^2\right) = 0. \tag{48}$$

$$\text{0-order:} \quad \hat{\mathcal{N}}(-i\partial_x) u_0 = 0 \quad \Rightarrow \quad (-i\partial_x \hat{\mathbf{x}}) \times u_0 = 0 \quad \Rightarrow \quad \partial_x (e_y^0, e_z^0, h_y^0, h_z^0) = 0 \tag{49}$$

$$\text{1-order:} \quad \underbrace{\hat{\mathcal{N}}(k_x) u_0}_{\perp \hat{\mathbf{x}}} - \omega \hat{\mathcal{M}} u_0 + \underbrace{\hat{\mathcal{N}}(-i\partial_x) \left. \frac{\partial u_{k_x}}{\partial k_x} \right|_{k_x=0} k_x}_{\perp \hat{\mathbf{x}}} = 0 \quad \Rightarrow \quad \left(\hat{\mathcal{M}} u_0\right) \cdot \hat{\mathbf{x}} = (d_x^0, b_x^0) = 0, \tag{50}$$

where $d_x^0, b_x^0$ denote the $x$ components of the 0-order $\mathbf{D}$ and $\mathbf{B}$ fields respectively. When $k_z = 0$, $\hat{U}$ is reduced to the identity matrix, thus $\widetilde{U}(k_z = 0) = \left(\hat{P}_- + \hat{G}\hat{P}_+\right)$ and $\widetilde{u}_0 = \widetilde{U}(k_z = 0) u_0 = \left(0, e_y^0, e_z^0, 0, h_y^0, h_z^0\right)$ is a transverse constant vector. So we obtian

$$\lim_{k_x \to 0} \widetilde{S}_{L/4} \Psi(k_x, 0, 0) = \widetilde{S}_{L/4} u_0 = \widetilde{U}^{-1} \hat{C}_{2x} \hat{T}_x(L/4) \widetilde{u}_0 = \widetilde{U}^{-1}(-\widetilde{u}_0) = -u_0. \tag{51}$$

Therefore, **both the two bands stemming from $|\mathbf{k}| = \omega = 0$ have the same branch index** $s = -1$.

### S5. Asymptotic dispersion of bands at infinity

For a general permitivitiy given by Eq. (1) of the main text, the wave equations of $\hat{M}_y$-odd and even modes on the $k_y = 0$ plane are, respectively,

$$\hat{M}_y\text{-odd:} \qquad \left[ -\varepsilon_{yy}^{-1/2} \frac{d^2}{dx^2} \varepsilon_{yy}^{-1/2} + k_z^2 \varepsilon_{yy}^{-1} \right] \left(\sqrt{\varepsilon_{yy}} E_y\right) = \frac{\omega^2}{c^2} \left(\sqrt{\varepsilon_{yy}} E_y\right) \tag{52}$$

$$\hat{M}_y\text{-even:} \qquad \left[ -\frac{d}{dx} \frac{\varepsilon_{xx}}{\varepsilon_1 \varepsilon_3} \frac{d}{dx} + k_z \left\{ -i \frac{d}{dx}, \frac{\varepsilon_{xz}}{\varepsilon_1 \varepsilon_3} \right\} + k_z^2 \frac{\varepsilon_{zz}}{\varepsilon_1 \varepsilon_3} \right] H_y = \frac{\omega^2}{c^2} H_y \tag{53}$$

where $\{\cdot, \cdot\}$ denotes the anticommutator. By introducing a new function $\widetilde{H}_y(x) = \hat{U} H_y = \exp\left(ik_z \int_0^x \frac{\varepsilon_{xz}(\xi)}{\varepsilon_{xx}(\xi)} d\xi\right) H_y$, Eq.(53) can be transformed into a standard Sturm-Liouville equation:

$$\left[ -\frac{d}{dx} \frac{\varepsilon_{xx}}{\varepsilon_1 \varepsilon_3} \frac{d}{dx} + k_z^2 \varepsilon_{xx}^{-1} \right] \widetilde{H}_y = \frac{\omega^2}{c^2} \widetilde{H}_y. \tag{54}$$

Therefore, the eigen-equations for $\hat{M}_y$ even and odd bands can be uniformly expressed as follows:

$$\left[ \hat{K} + k_z^2 V(x) \right] \psi = \frac{\omega^2}{c^2} \psi, \tag{55}$$

with Bloch boundary condition $\psi(0) = e^{ik_x L} \psi(L)$, $\frac{d}{dx} \psi(0) = e^{ik_x L} \frac{d}{dx} \psi(L)$, where $\hat{K}$ is a positive semidefinite operator arising from the fact that the permittivity tensor of dielectrics is positive definite, and $V(x) = V(x + L)$ is a positive piecewise smooth function (so it has infimum $\inf(V) = V_{\min}$).

**Theorem 1.** *All bands $\omega_n(k_z)$ of Eq.(55) tend to a unique asymptotic linear dispersion, as $k_z \to \infty$, and the asymptotic slope is determined by the infimum of $V(x)$:*

$$\lim_{k_z\to\infty}\frac{\omega_n}{k_z} = \lim_{k_z\to\infty}\frac{d\omega_n}{dk_z} = c\sqrt{V_{\min}}. \tag{56}$$

*Proof.* 1) Lower bound: left product of $\psi_n(x)$ to Eq.(55) yields $\omega_n^2/c^2 = \langle\psi_n|\hat{K}|\psi_n\rangle + k_z^2\langle\psi_n|V|\psi_n\rangle \geq k_z^2 V_{\min}$, since $\hat{K}$ is positive semidefinite. Hence, we have $\frac{\omega_n}{k_z} \geq c\sqrt{V_{\min}}$.

2) Upper bound: for a fixed $k_z$, all the eigenstates $\{\psi_i\}$ of Eq.(55) from a compete basis of the function space $F$ satisfying the boundary conditions. And we consider the subspace $\mathrm{span}\{\psi_1,\cdots,\psi_{n-1}\}$ spanned by the eigensates corresponding to the first $n-1$ eigenvalues $\omega_1^2 \leq \cdots \leq \omega_{n-1}^2 \leq \omega_n^2 \leq \cdots$, and its complement space is $F\backslash\mathrm{span}\{\psi_1,\cdots\psi_{n-1}\} = \mathrm{span}\{\psi_n,\psi_{n+1},\cdots\}$.

On the other hand, $\forall\epsilon > 0$, we can find $n$-D function space $F_n$ satisfying the boundary conditions, such that $\frac{\langle u|V|u\rangle}{\langle u|u\rangle} \leq V_{\min} + \epsilon$, $\forall u(x) \not\equiv 0 \in F_n$. And since $\dim F_n = n > \dim\mathrm{span}\{\psi_1,\cdots,\psi_{n-1}\}$, there exists a state $v(x) \neq 0 \in F_n \cap \mathrm{span}\{\psi_n,\psi_{n+1},\cdots\}$, so we can expand $v(x)$ using the eigenstates $\psi_i$ $(i \geq n)$: $v(x) = \sum_{i=n}^{\infty}\alpha_i\psi_i(x)$, and accordingly

$$\frac{\omega_n^2}{c^2} \leq \frac{\sum_{i=n}^{\infty}|\alpha_i|^2\omega_i^2/c^2}{\sum_{i=n}^{\infty}|\alpha_i|^2} = \frac{\langle v|\hat{K}+k_z^2V|v\rangle}{\langle v|v\rangle} \leq K_{\max} + k_z^2(V_{\min}+\epsilon),$$

where $K_{\max} = \max\left\{\frac{\langle v|\hat{K}|v\rangle}{\langle v|v\rangle}\middle| u(x) \neq 0 \in F_n\right\} < \infty$ is the upper bound of $\hat{K}$ in the subspace $F_n$. Therefore, we have

$$\frac{\omega_n}{c\,k_z} \leq \inf_{\epsilon>0}\sqrt{\frac{K_{\max}}{k_z^2} + V_{\min} + \epsilon}.$$

As $k_z \to \infty$, both the lower and upper bounds of $\omega_n/k_z$ tend to $\inf_{\epsilon>0}\sqrt{V_{\min}+\epsilon} = \sqrt{V_{\min}}$, so we obtain the limit $\lim_{k_z\to\infty}\omega_n/k_z = c\sqrt{V_{\min}}$. And due to L'Hôpital's rule, we also have $\lim_{k_z\to\infty}d\omega/dk_z = c\sqrt{V_{\min}}$. □

**Remark**: The theorem can also be proved using min-max theorem of eigenvalues [2]. Indeed, some procedures in the proof of min-max theorem have also been used in the above proof. In addition, Ref. [3] offers a different proof.

As a consequence, the asymptotic group velocities of even bands and order bands are, respectively,

$$\begin{aligned}\lim_{k_z\to\infty}\frac{d\omega_n^{\mathrm{odd}}}{dk_z} &= \frac{c}{\sqrt{\varepsilon_{yy}^{\max}}},\\ \lim_{k_z\to\infty}\frac{d\omega_n^{\mathrm{even}}}{dk_z} &= \frac{c}{\sqrt{\varepsilon_{xx}^{\max}}}.\end{aligned} \tag{57}$$

This asymptotic group velocities are numerically verified, as shown in Fig. 2d of the main text. Therefore, along $\Gamma - Z$ direction, all bands tend to a linear dispersion as $k_z \to \infty$. All even and all odd bands have identical asymptotic group velocities respectively. For almost any dielectric photonic crystal respecting the symmetries, there exits a pair of triple points as the nexus of the nodal lines (intersection either of 1st even and 2nd,3rd odd bands or of 1st odd and 2nd,3rd even bands). The only exception (zero measure) occurs when the asymptotic group velocities of even and odd bands are identical, namely $\varepsilon_{xx}^{\max} = \varepsilon_{yy}^{\max}$.

## S6. Robustness of nexus points against the variation of materials

In the main text, we have expounded that the special photonic band connectivity induced by the hidden symmetry guarantees that the triply degenerate NPs can almost always emerge on the 4 lowest bands along $\Gamma - Z$. In Fig. S1, we use the biaxial dielectrics to construct the AB-layer-stacked PhCs and fixed the values of $\varepsilon_{xx}$, $\varepsilon_{zz}$, and $\varepsilon_{xz}$ for the PhCs in all the panels, while we change the value of $\varepsilon_{yy}$ in each panel to show how the nodal structure changes with the parameter of the PhC and to demonstrate the robustness of NPs. We note that there are infinite NLs in the band structures, while only those connecting with the lowest triple NPs are plotted.

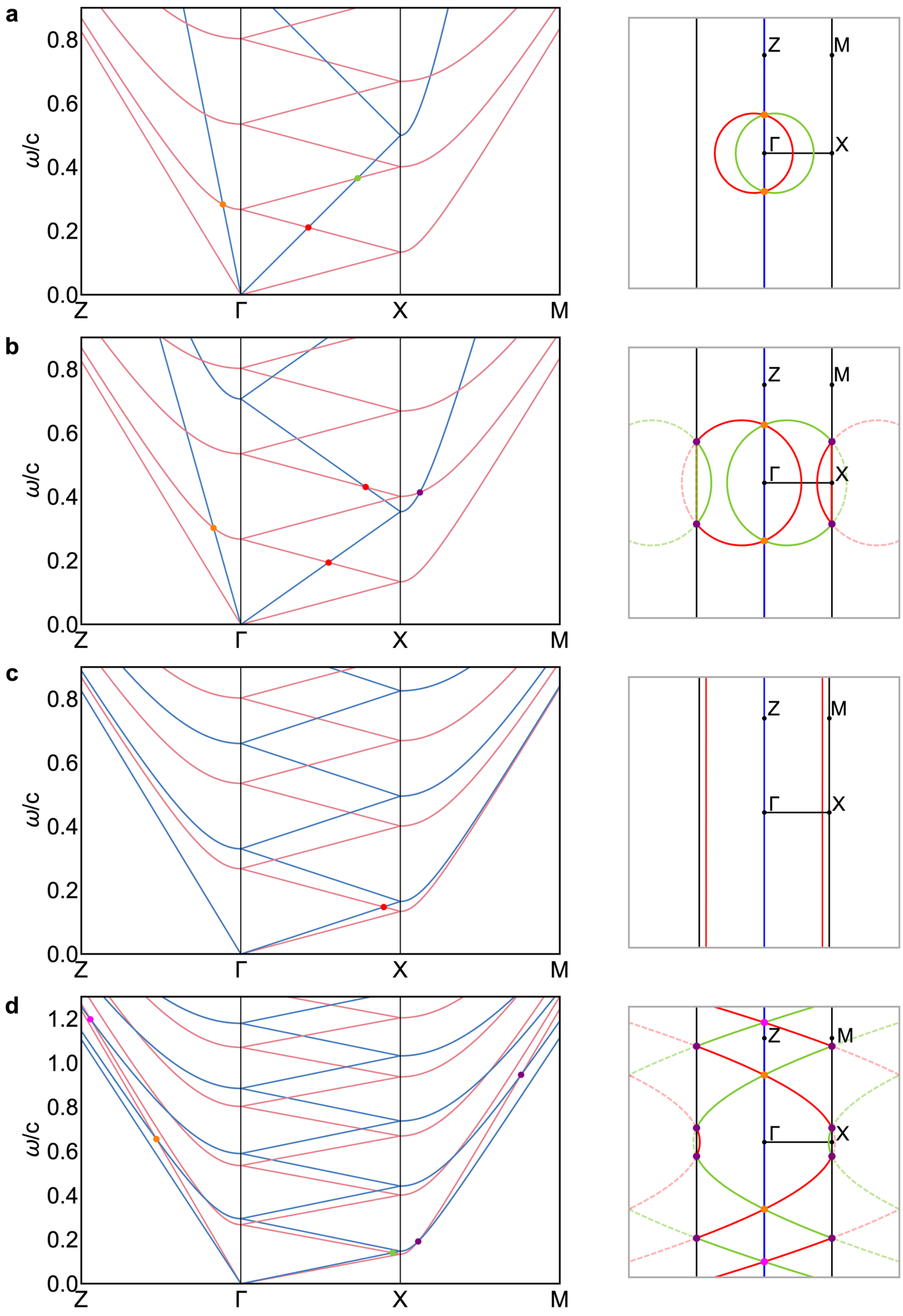


**Figure S1**. The band structures and nodal structures on the $k_y = 0$ plane for the AB-layered PhCs with fixed parameters $\varepsilon_{xx} = 9.17$, $\varepsilon_{zz} = 14.83$, $|\varepsilon_{xz}| = 2.83$, and with different values of $\varepsilon_{yy}$ in each panel: **a**. $\varepsilon_{yy} = 1$; **b**. $\varepsilon_{yy} = 2$; **c**. $\varepsilon_{yy} = \varepsilon_{xx} = 9.17$; **d**. $\varepsilon_{yy} = 11.5$. The light blue and light red lines denote $\hat{M}_y$-odd and $\hat{M}_y$-even bands, respectively. Red and green dots and curves denote nodal lines protected by mirror and $\mathcal{PT}$ symmetries. Blue lines denote Kramers-like nodal lines. Orange dots denote triply degenerate nexus points. Purple and magenta dots denote fourfold degenerate nexus points.

In Fig. S1a, we let $\varepsilon_{yy} < \varepsilon_{xx}$. As such, the asymptotic group velocity of $\hat{M}_y$-odd bands (light blue) is larger than that of $\hat{M}_y$-even bands (light red) in the $\Gamma - Z$ direction. So the lowest triply degenerate NPs (orange dots) are formed by the $1^{\text{st}}$ odd and $2^{\text{nd}}$, $3^{\text{rd}}$ even bands at the intersections of two nodal rings (red and green) and the lowest Kramers-like NL (blue).

If we increase the value of $\varepsilon_{yy}$ while keeping it less than $\varepsilon_{xx}$, the two nodal rings will grow bigger and will eventually intersect with their transnational counterparts at the fourfold degenerate NPs (purple dots) on the two boundaries of the BZ as shown in Fig. S1b. As a result, the nodal rings in the extended BZ connect together and form a nodal chain in the $k_y = 0$ plane.

As $\varepsilon_{yy}$ increases, the eccentricity of the two nodal rings grows accordingly, and the two triple NPs move outward along the $z$ axis from the origin. When $\varepsilon_{yy}$ reaches the critical value $\varepsilon_{yy} = \varepsilon_{xx}$, the asymptotic group velocities of even and odd bands are accidentally identical in the $z$ direction. Then, the two NPs move to the infinity and vanish, and the two nodal rings are reduced to two straight lines parallel to $\Gamma - Z$, as shown in Fig. S1c. As we have emphasized in the main text, the two triple NPs in the lowest 4 bands can only disappear in this accidental condition of $\varepsilon_{yy} = \varepsilon_{xx}$. However, if we arbitrarily select the parameters of the PhCs respecting the symmetries, the probability of encountering these exceptional cases is zero, since they are restricted to a subset of measure zero for all possible parameters.

Once $\varepsilon_{yy}$ is larger than $\varepsilon_{xx}$, Fig. S1d demonstrates that the two triple NPs will reappear immediately. However, in this case, the two NPs are on the band crossings of $1^{\text{st}}$ even and $2^{\text{nd}}$,$3^{\text{rd}}$ odd bands, as the asymptotic group velocity of even bands surpasses that of odd bands. At the same time, Fig. S1d shows that the two previous ring shaped nodal lines convert to two hyperbolas. And the nodal hyperbolas intersect with their translational counterparts infinite times on the $\Gamma - Z$ line as well as on the two BZ boundaries, forming fourfold degenerate NPs (magenta dots and purple dots).

In Fig. S2, we plot the band structure near a typical fourfold degenerate NP on the BZ boundary ($X - M$). As shown in Fig. S2a,b, there are 4 nodal rings intersect on the NPs. In the section of $k_x = \pi/L$, there are two doubly degenerate bands intersecting at the NP as displayed in Fig. S2c, resulting from the combined symmetry of time reversal and twofold screw rotation $\hat{\Theta}_{L/2} = \mathcal{T}\hat{S}_{2x}$. Furthermore, Fig. S2d,e show that both the band structure in the $k_z = k_z^{\text{NP}}$ section and the iso-frequency surfaces at $\omega^{\text{NP}}$ disperse as 2D double Dirac cones [4].

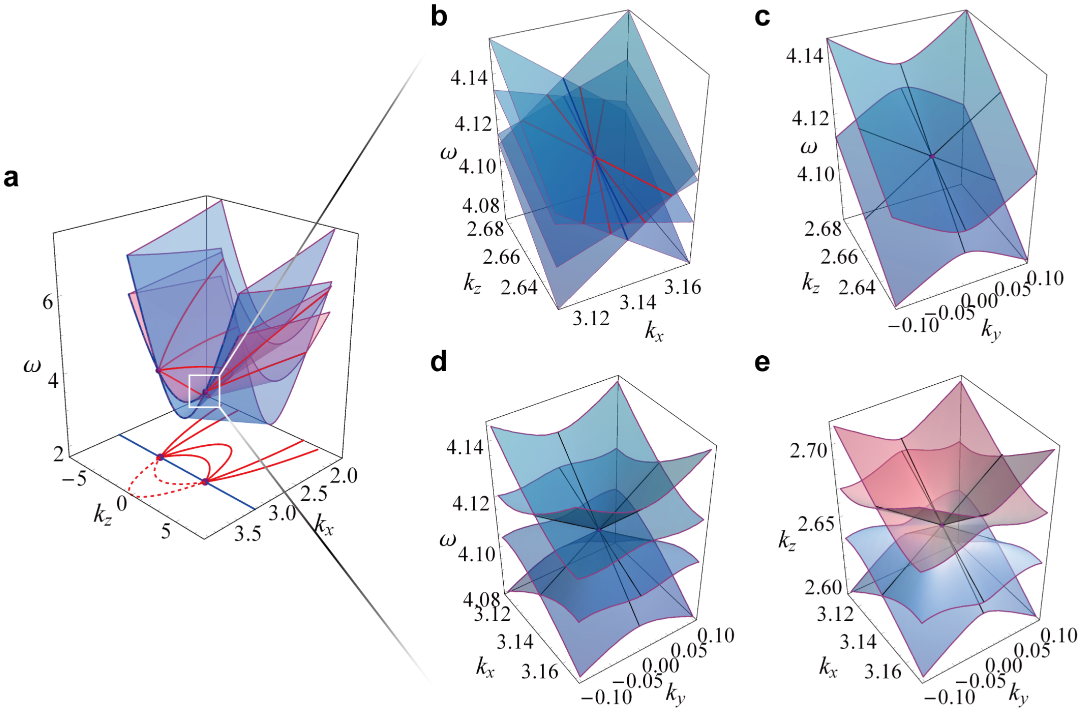


**Figure S2**. Band structures near a fourfold degenerate nexus point in a AB-layer-stacked PhC with parameters $\varepsilon_1 = \varepsilon_2 = 1$, $\varepsilon_3 = 12$ and $\theta = \pi/12$. **a**. Band structure near the nexus of 4 nodal lines on the $k_y = 0$ plane. **b-d**, Zoomed in band structure near the NP in the sections of $k_y = 0$, $k_x = \pi/L$, and $k_z = k_z^{\text{NP}}$, respectively. **e**. Iso-frequency surfaces near the NP at the frequency of the NP.

### S7. Derivation of $\mathbf{k}\cdot\mathbf{p}$ Hamiltonian for layer-stacked photonic crystals

Here we introduce a general $\mathbf{k}\cdot\mathbf{p}$ framework for a generic layer-stacked photonic crystal that is periodic in $x$ direction and homogeneous in the other two dimensions $\mathbf{r}_\perp = (y, z)$. In comparison with using wave equation (a 2-order PDE), it is more convenient to perform the derivation directly from the Maxwell's equations (1-order PDEs):

$$\underbrace{\begin{pmatrix} 0 & i\nabla\times \\ -i\nabla\times & 0 \end{pmatrix}}_{\hat{\mathcal{N}}} \underbrace{\begin{pmatrix} \mathbf{E} \\ \mathbf{H} \end{pmatrix}}_{\Psi} = \omega \underbrace{\begin{pmatrix} \overleftrightarrow{\varepsilon}(x) & \overleftrightarrow{\chi}(x) \\ \overleftrightarrow{\chi}(x)^\dagger & \overleftrightarrow{\mu}(x) \end{pmatrix}}_{\hat{\mathcal{M}}} \begin{pmatrix} \mathbf{E} \\ \mathbf{H} \end{pmatrix}, \tag{58}$$

Expending of the Bloch state $\Psi_{n,\mathbf{k}}(\mathbf{r}) = e^{i\mathbf{k}\cdot\mathbf{r}} u_{n,\mathbf{k}}(x) = e^{i\mathbf{k}_\perp\cdot\mathbf{r}_\perp} \widetilde{\Psi}_{n,\mathbf{k}}(x)$ using the states at $\mathbf{k}_0$ (usually a degenerate point), we obtain

$$\Psi_{n,\mathbf{k}} = \sum_m A_{nm}(\mathbf{k}) e^{i(\mathbf{k}-\mathbf{k}_0)\cdot\mathbf{r}} \Psi_{m,\mathbf{k}_0}. \tag{59}$$

Substitution of the expansion into Eq. (58) yields

$$\begin{aligned} 0 &= \left(\hat{\mathcal{N}} - \omega_{n,\mathbf{k}}\hat{\mathcal{M}}\right)\Psi_{n\mathbf{k}} = \sum_m \left[\hat{\mathcal{N}} e^{i(\mathbf{k}-\mathbf{k}_0)\cdot\mathbf{r}} \Psi_{m,\mathbf{k}_0} - \omega_{n,\mathbf{k}} \hat{\mathcal{M}} e^{i(\mathbf{k}-\mathbf{k}_0)\cdot\mathbf{r}} \Psi_{m,\mathbf{k}_0}\right] A_{nm} \\ &= e^{i(\mathbf{k}-\mathbf{k}_0)\cdot\mathbf{r}} \sum_m \left[\begin{pmatrix} 0 & (\mathbf{k}_0-\mathbf{k})\times \\ -(\mathbf{k}_0-\mathbf{k})\times & 0 \end{pmatrix} + \left(\omega_{m,\mathbf{k}_0} - \omega_{n,\mathbf{k}}\right)\hat{\mathcal{M}}\right] \Psi_{m,\mathbf{k}_0} A_{nm}. \end{aligned} \tag{60}$$

Since the Brillouin zone of a layer-stacked PhC is infinite in the transverse plane, the orthogonality of the Bloch states should be written as

$$\left\langle \Psi_{n,\mathbf{k}} \middle| \hat{\mathcal{M}} \middle| \Psi_{m,\mathbf{k}'} \right\rangle = \frac{1}{(2\pi)^2} \int_{-\infty}^{\infty} e^{i(\mathbf{k}'_\perp - \mathbf{k}_\perp)\cdot\mathbf{r}_\perp} d\mathbf{r}_\perp \int_{-L/2}^{L/2} dx \widetilde{\Psi}^\dagger_{n,\mathbf{k}} \hat{\mathcal{M}} \widetilde{\Psi}_{m,\mathbf{k}'} = \delta(\mathbf{k}'_\perp - \mathbf{k}_\perp)\delta_{nm}\delta_{k_x,k'_x}, \tag{61}$$

where $\mathbf{k}_\perp$ and $\mathbf{r}_\perp$ are transverse wavevector and position vector respectively, $\delta(\mathbf{k}'_\perp - \mathbf{k}_\perp)$ represents Dirac delta function, while the other two delta functions are Kronecker delta. Performing inner product between $\Psi_{m',\mathbf{k}_0}$ and Eq. (60), we obtain the $\mathbf{k}\cdot\mathbf{p}$ eigen-equation of the 1$^{\text{st}}$-order

$$\sum_m \left[\int_{-L/2}^{L/2} dx \Psi^\dagger_{m',\mathbf{k}_0} \begin{pmatrix} 0 & (\mathbf{k}_0-\mathbf{k})\times \\ -(\mathbf{k}_0-\mathbf{k})\times & 0 \end{pmatrix} \Psi_{m,\mathbf{k}_0} + \left(\omega_{m,\mathbf{k}_0} - \omega_{n,\mathbf{k}}\right)\delta_{m'm}\right] A_{nm} = 0, \tag{62}$$

which can be rewritten as

$$\sum_m H_{m'm} A_{nm} = \left(\omega_{n,\mathbf{k}} - \omega_{m',\mathbf{k}_0}\right) A_{nm'}, \tag{63}$$

with the element of the 1$^{\text{st}}$-order $\mathbf{k}\cdot\mathbf{p}$ Hamiltonian

$$H_{m'm} = (\mathbf{k}-\mathbf{k}_0)\cdot \int_{-L/2}^{L/2} dx \left[\mathbf{E}_{m,\mathbf{k}_0} \times \mathbf{H}^*_{m',\mathbf{k}_0} - \mathbf{H}_{m,\mathbf{k}_0} \times \mathbf{E}^*_{m',\mathbf{k}_0}\right] = (\mathbf{k}-\mathbf{k}_0)\cdot\mathbf{p}_{m'm}. \tag{64}$$

#### A. $\mathbf{k}\cdot\mathbf{p}$ Hamiltonian near the nexus points

At the triply degenerate NPs with $\mathbf{k}^{\text{NP}_\pm} = (0, 0, \pm\frac{2\pi}{L}\sqrt{\frac{\varepsilon_{xx}}{\varepsilon_{yy}-\varepsilon_{xx}}})$ and $\omega^{\mathbf{NP}} = \frac{2c\pi}{L\sqrt{\varepsilon_{yy}-\varepsilon_{xx}}}$ for the AB-layer-stacked PhC in Fig. 1 of the main text, the three degenerate eigenstates can be obtained from Eqs. (13) and (14):

$$\Psi_1^{\text{odd}}(\mathbf{k}^{\text{NP}_\pm}) = (\mathbf{E}_1, \mathbf{H}_1) = \frac{1}{\sqrt{2L}} \left(0, 1, 0, \mp\sqrt{\frac{\varepsilon_{xx}}{\varepsilon_{yy}}}, 0, \sqrt{\frac{\varepsilon_{yy}-\varepsilon_{xx}}{\varepsilon_{yy}}}\right)^\top \exp\left[i\left(\frac{2\pi}{L}x + k_z^{\text{NP}_\pm} z\right)\right], \tag{65}$$

$$\Psi_0^{\text{even}}(\mathbf{k}^{\text{NP}_\pm}) = (\mathbf{E}_2, \mathbf{H}_2) = \frac{1}{\sqrt{2L}} \left(\frac{1}{\varepsilon_{xx}}, 0, 0, 0, 1, 0\right)^\top \exp\left[ik_z^{\text{NP}_\pm}\left(-\frac{\varepsilon_{xz}}{\varepsilon_{xx}}x + z\right)\right], \tag{66}$$

$$\Psi_{-1}^{\text{odd}}(\mathbf{k}^{\text{NP}_\pm}) = (\mathbf{E}_3, \mathbf{H}_3) = \frac{1}{\sqrt{2L}} \left(0, 1, 0, \mp\sqrt{\frac{\varepsilon_{xx}}{\varepsilon_{yy}}}, 0, -\sqrt{\frac{\varepsilon_{yy}-\varepsilon_{xx}}{\varepsilon_{yy}}}\right)^\top \exp\left[i\left(-\frac{2\pi}{L}x + k_z^{\text{NP}_\pm} z\right)\right]. \tag{67}$$

Substituting Eqs. (65)-(67) into Eq. (64), we then obtain the 1st-order $\mathbf{k}\cdot\mathbf{p}$ Hamiltonian at the NPs:

$$\hat{H}^{\pm\prime}_{\mathrm{NP}} = \begin{pmatrix} v_x\delta k_x \pm v_z^{\mathrm{odd}}\delta k_z & \dfrac{-i\,v_{y0}^*}{\sqrt{2}}\delta k_y & 0 \\ \dfrac{i\,v_{y0}}{\sqrt{2}}\delta k_y & \pm v_z^{\mathrm{even}}\delta k_z & \dfrac{-i\,v_{y0}}{\sqrt{2}}\delta k_y \\ 0 & \dfrac{i\,v_{y0}^*}{\sqrt{2}}\delta k_y & -v_x\delta k_x \pm v_z^{\mathrm{odd}}\delta k_z \end{pmatrix}, \tag{68}$$

where $\delta\mathbf{k} = (\delta k_x, \delta k_y, \delta k_z) = \mathbf{k} - \mathbf{k}^{\mathrm{NP}_\pm}$, and

$$v_x = \frac{\sqrt{\varepsilon_{yy}-\varepsilon_{xx}}}{\varepsilon_{yy}}, \quad v_{y0} = \frac{\varepsilon_{xz}(\varepsilon_{yy}-\varepsilon_{xx})}{\pi\sqrt{2\varepsilon_{yy}}(\varepsilon_{xz}^2 - \varepsilon_{xx}\varepsilon_{yy} + \varepsilon_{xx}^2)}\left(1+\exp\left(\frac{\mp i\varepsilon_{xz}\pi}{\sqrt{\varepsilon_{xx}(\varepsilon_{yy}-\varepsilon_{xx})}}\right)\right), \quad v_z^{\mathrm{odd}} = \frac{1}{\sqrt{\varepsilon_{xx}}}, \quad v_z^{\mathrm{even}} = \frac{\sqrt{\varepsilon_{xx}}}{\varepsilon_{yy}}. \tag{69}$$

Under the unitary transformation $\hat{V} = \mathrm{diag}(1, v_{y0}^*/|v_{y0}|, 1)$, the Hamitonian of the NPs converts to

$$\hat{H}^{\pm}_{\mathrm{NP}} = \hat{V}\hat{H}^{\pm\prime}_{\mathrm{NP}}V^\dagger = \begin{pmatrix} v_x\delta k_x \pm v_z^{\mathrm{odd}}\delta k_z & \dfrac{-i\,v_y}{\sqrt{2}}\delta k_y & 0 \\ \dfrac{i\,v_y}{\sqrt{2}}\delta k_y & \pm v_z^{\mathrm{even}}\delta k_z & \dfrac{-i\,v_y}{\sqrt{2}}\delta k_y \\ 0 & \dfrac{i\,v_y}{\sqrt{2}}\delta k_y & -v_x\delta k_x \pm v_z^{\mathrm{odd}}\delta k_z \end{pmatrix} \tag{70}$$

with $v_y = |v_{y0}|$. In general, a $3\times3$ Hermitian matrix can be expanded by the 8 generators of $\mathsf{su}(3)$ Lie algebra. The 8 generators are usually selected as the 8 Gell-Mann matrices, or alternatively selected as the 3 spin-1 operators together with 5 spin-1 quadrupolar operators [5, 6]. Here, in order to exhibit the relation between the nexus points and spin-1 physics, we adopt the latter choice, so Eq. (70) can be rewritten as

$$\hat{H}^{\pm}_{\mathrm{NP}} = v_x\hat{S}_z\delta k_x + v_y\hat{S}_y\delta k_y \pm \left[q_z\hat{Q}_{zz} + v_{z0}\hat{I}\right]\delta k_z, \tag{71}$$

where $q_z = v_z^{\mathrm{odd}} - v_z^{\mathrm{even}}$, $v_{z0} = \frac{1}{3}(2v_z^{\mathrm{odd}} + v_z^{\mathrm{even}})$, $\hat{S}_i$ $(i = x,y,z)$ denote the 3 spin-1 operators, and $\hat{Q}_{zz}$ denotes one of the spin-1 quadrupolar operator [5, 6], which take the forms

$$\hat{S}_x = \frac{1}{\sqrt{2}}\begin{pmatrix}0&1&0\\1&0&1\\0&1&0\end{pmatrix}, \quad \hat{S}_y = \frac{1}{\sqrt{2}}\begin{pmatrix}0&-i&0\\i&0&-i\\0&i&0\end{pmatrix}, \quad \hat{S}_z = \begin{pmatrix}1&0&0\\0&0&0\\0&0&-1\end{pmatrix}, \quad \hat{Q}_{zz} = (\hat{S}_z)^2 - \frac{1}{3}\sum_i(\hat{S}_i)^2 = \frac{1}{3}\begin{pmatrix}1&0&0\\0&-2&0\\0&0&1\end{pmatrix}. \tag{72}$$

## S8. Spin-1 conical diffraction

According to the $\mathbf{k}\cdot\mathbf{p}$ Hamiltonian near an NP, e.g. $\mathbf{k}^{\mathrm{NP}_+}$, the eigen-equation for states on the iso-frequency surface at the frequency $\omega^{\mathrm{NP}}$, *i.e.* $\delta\omega = 0$, reads

$$\underbrace{\begin{pmatrix} v_x\delta k_x & \dfrac{-i\,v_y}{\sqrt{2}}\delta k_y & 0 \\ \dfrac{i\,v_y}{\sqrt{2}}\delta k_y & 0 & \dfrac{-i\,v_y}{\sqrt{2}}\delta k_y \\ 0 & \dfrac{i\,v_y}{\sqrt{2}}\delta k_y & -v_x\delta k_x \end{pmatrix}}_{\widetilde{H}(\delta\mathbf{k}_{xy}) = v_x\hat{S}_z\delta k_x + v_y\hat{S}_y\delta k_y}\widetilde{\psi} = -\delta k_z\begin{pmatrix} v_z^{\mathrm{odd}} & 0 & 0 \\ 0 & v_z^{\mathrm{even}} & 0 \\ 0 & 0 & v_z^{\mathrm{odd}} \end{pmatrix}\widetilde{\psi}. \tag{73}$$

Using the transformation $\hat{R} = \mathrm{diag}(1, \sqrt{v_z^{\mathrm{even}}/v_z^{\mathrm{odd}}}, 1)$ and replacing $\delta k_z \to -i\partial_z$, we obtain the effective Schrödinger equation for the states on the iso-frequency surface:

$$i\,v_z^{\mathrm{odd}}\frac{\partial}{\partial z}|\psi\rangle = \hat{H}(\delta\mathbf{k}_{xy})|\psi\rangle, \tag{74}$$

with the eigenstate $|\psi\rangle = \hat{R}\widetilde{\psi}$, and the effective anisotropic 2D spin-1 Hamiltonian:

$$\hat{H}(\delta\mathbf{k}_{xy}) = \hat{R}^{-1}\widetilde{H}(\delta\mathbf{k}_{xy})\hat{R}^{-1} = \begin{pmatrix} v_x\delta k_x & \frac{-i\,\tilde{v}_y}{\sqrt{2}}\delta k_y & 0 \\ \frac{i\,\tilde{v}_y}{\sqrt{2}}\delta k_y & 0 & \frac{-i\,\tilde{v}_y}{\sqrt{2}}\delta k_y \\ 0 & \frac{i\,\tilde{v}_y}{\sqrt{2}}\delta k_y & -v_x\delta k_x \end{pmatrix} = v_x\hat{S}_z\delta k_x + \tilde{v}_y\hat{S}_y\delta k_y, \tag{75}$$

where $\tilde{v}_y = \sqrt{v_z^{\text{even}}/v_z^{\text{odd}}}v_y = \sqrt{\varepsilon_{xx}/\varepsilon_{yy}}v_y$. In addition, we can introduce the spin-1 ladder operators in $\hat{S}_x$ representation:

$$\hat{S}_\pm = \hat{S}_z \mp i\hat{S}_y = \begin{pmatrix} 1 & \mp 1/\sqrt{2} & 0 \\ \pm 1/\sqrt{2} & 0 & \mp 1/\sqrt{2} \\ 0 & \pm 1/\sqrt{2} & 1 \end{pmatrix}, \tag{76}$$

which raises and lowers a spin quantum number of the eigenstates of $\hat{S}_x$, respectively,

$$\hat{S}_\pm |s\rangle = \sqrt{2 - s(s \pm 1)}\,|s \pm 1\rangle\,, \tag{77}$$

where $|s\rangle$ represent an eigenstate of $\hat{S}_x$ with spin quantum number (eigenvalue) $s$, *i.e.* $\hat{S}_x|s\rangle = s|s\rangle$ ($s \in \{-1, 0, 1\}$). As such, the effective spin-1 Hamiltonian (75) can be rewritten using the ladder operators:

$$\hat{H}(\delta\mathbf{k}_{xy}) = \frac{\delta\tilde{k}(\delta\mathbf{k}_{xy})}{2}\left(e^{i\phi(\delta\mathbf{k}_{xy})}\hat{S}_+ + e^{-i\phi(\delta\mathbf{k}_{xy})}\hat{S}_-\right), \tag{78}$$

with $\delta\tilde{k}(\delta\mathbf{k}_{xy})$ and $\phi(\delta\mathbf{k}_{xy})$ denoting the modulus and argument of $\delta\tilde{k}e^{i\phi} = v_x\delta k_x + i\tilde{v}_y\delta k_y$. Moreover, the evolution of a state along the $z$ axis can be explicitly expressed using the evolution operator:

$$|\psi(z)\rangle = \exp\left[\frac{-iz}{v_z^{\text{odd}}}\hat{H}(\delta\mathbf{k}_{xy})\right]|\psi_0\rangle = \left\{I + \sum_{n=1}^{\infty}\frac{1}{n!}\left[\frac{-iz}{v_z^{\text{odd}}}\frac{\delta\tilde{k}}{2}\left(e^{i\phi}\hat{S}_+ + e^{-i\phi}\hat{S}_-\right)\right]^n\right\}|\psi_0\rangle \tag{79}$$

where $|\psi_0\rangle$ represents the input state at $z = 0$. Using the ladder operators, we can obtain the following formulas:

$$\hat{h}^n|0\rangle = \begin{cases} \frac{2^n}{\sqrt{2}}\left(e^{i\phi}|1\rangle + e^{-i\phi}|-1\rangle\right), & (n \in \text{odd}) \\ 2^n|0\rangle, & (n \in \text{even}), \end{cases} \quad \text{and} \quad \hat{h}^n|\pm 1\rangle = \sqrt{2}e^{\mp i\phi}\left(\hat{h}^{n-1}|0\rangle\right), \tag{80}$$

with $\hat{h} = e^{i\phi}\hat{S}_+ + e^{-i\phi}\hat{S}_-$. Using these formulas, we can derive the final states evolving from different eigenstates of $\hat{S}_x$ as input:

$$\begin{aligned} \exp\left[\frac{-iz}{v_z^{\text{odd}}}\hat{H}(\delta\mathbf{k}_{xy})\right]|1\rangle &= \frac{1}{2}\left[\cos\left(\frac{\delta\tilde{k}\,z}{v_z^{\text{odd}}}\right) + 1\right]|1\rangle - \frac{i}{\sqrt{2}}\sin\left(\frac{\delta\tilde{k}\,z}{v_z^{\text{odd}}}\right)e^{-i\phi}|0\rangle + \frac{1}{2}\left[\cos\left(\frac{\delta\tilde{k}\,z}{v_z^{\text{odd}}}\right) - 1\right]e^{-2i\phi}|-1\rangle\,, \\ \exp\left[\frac{-iz}{v_z^{\text{odd}}}\hat{H}(\delta\mathbf{k}_{xy})\right]|0\rangle &= \cos\left(\frac{\delta\tilde{k}\,z}{v_z^{\text{odd}}}\right)|0\rangle - \frac{i}{\sqrt{2}}\sin\left(\frac{\delta\tilde{k}\,z}{v_z^{\text{odd}}}\right)\left(e^{i\phi}|1\rangle + e^{-i\phi}|-1\rangle\right), \\ \exp\left[\frac{-iz}{v_z^{\text{odd}}}\hat{H}(\delta\mathbf{k}_{xy})\right]|-1\rangle &= \frac{1}{2}\left[\cos\left(\frac{\delta\tilde{k}\,z}{v_z^{\text{odd}}}\right) + 1\right]|-1\rangle - \frac{i}{\sqrt{2}}\sin\left(\frac{\delta\tilde{k}\,z}{v_z^{\text{odd}}}\right)e^{i\phi}|0\rangle + \frac{1}{2}\left[\cos\left(\frac{\delta\tilde{k}\,z}{v_z^{\text{odd}}}\right) - 1\right]e^{2i\phi}|1\rangle\,. \end{aligned} \tag{81}$$

If we project the final state onto an spin eigenstate $|s_f\rangle$ of $\hat{S}_x$, we obtain the compact form of the transition amplitude from an input spin state $|s_i\rangle$ to the output state $|s_f\rangle$:

$$\langle s_f|\exp\left[\frac{-iz}{v_z^{\text{odd}}}\hat{H}(\delta\mathbf{k}_{xy})\right]|s_i\rangle = \exp\left[i\left(s_f - s_i\right)\phi(\delta\mathbf{k}_{xy})\right]\left(\frac{1}{2}\right)^{\frac{s_f^2 + s_i^2}{2}}\left[i^{|s_f - s_i|}\cos\left(\frac{\delta\tilde{k}(\delta\mathbf{k}_{xy})}{v_z^{\text{odd}}}z + \frac{\pi}{2}|s_f - s_i|\right) + s_i s_f\right]. \tag{82}$$

The result reveals that the phase of the output field winds $l = (s_f - s_i)$ times around $\delta\mathbf{k}_{xy} = 0$. Correspondingly, the phase on the ring of conical diffraction in the real space forms an optical vortex carrying the charge $l = (s_f - s_i) \in \{0, \pm 1, \pm 2\}$.

Indeed, the equality of the charge of the generated optical vortex and the difference of the spin quantum number of finial and initial states reflects the conservation of the generalized total angular momentum during the diffraction process. Since the effective 2D spin-1 Hamiltonian (75) has anisotropic Fermi velocity in the $xy$ plane, the total angular momentum is not a conserved quantity of the Hamiltonian. Nevertheless, we can rewrite the Hamiltonian as

$$\hat{H}(\delta\mathbf{k}_{xy}) = v_x \hat{S}^z \delta k_x + \tilde{v}_y \hat{S}^y \delta k_y = {e_a}^i \hat{S}^a \delta k_i, \qquad (a = z, y,\ i = x, y) \tag{83}$$

with regarding the anisotropic Fermi velocity tensor as the tetard $\left({e_a}^i\right) = \overleftrightarrow{v}_f = \mathrm{diag}(v_x, \tilde{v}_y)$ of an anisotropic space. The corresponding metric tensor of the space is given by $\left(g^{ij}\right) = \left(\delta^{ab}{e_a}^i{e_b}^j\right) = \mathrm{diag}(v_x^2, \tilde{v}_y^2)$. Then we can define a generalized orbital angular momentum operator for the anisotropic space:

$$\widetilde{L}_z = \frac{1}{\sqrt{\det(g^{ij})}} \epsilon_{zij} r^i \delta k^j = \frac{1}{\sqrt{\det(g^{ij})}} \epsilon_{zij} r^i g^{jl} \delta k_l = \frac{\tilde{v}_y}{v_x} x \delta k_y - \frac{v_x}{\tilde{v}_y} y \delta k_x. \tag{84}$$

where $\epsilon_{zij}$ denotes the antisymmetric symbol, $(r^i) = (x, y)$. In terms of the coordinate transformation $\delta\tilde{k}e^{i\phi} = v_x\delta k_x + i\tilde{v}_y\delta k_y$ where $\tilde{k}$,$\phi$ can be viewed as the generalized polar coordinates in the momentum space, we have

$$\frac{\partial}{\partial\phi} = \frac{\partial\,\delta k_x}{\partial\phi}\frac{\partial}{\partial\delta k_x} + \frac{\partial\,\delta k_y}{\partial\phi}\frac{\partial}{\partial\delta k_y} = \left(\frac{-\tilde{v}_y}{v_x}\delta k_y\right)\frac{\partial}{\partial\delta k_x} + \left(\frac{v_x}{\tilde{v}_y}\delta k_x\right)\frac{\partial}{\partial\delta k_y} = i\frac{\tilde{v}_y}{v_x}\left(i\frac{\partial}{\partial\delta k_x}\right)\delta k_y - i\frac{v_x}{\tilde{v}_y}\left(i\frac{\partial}{\partial\delta k_y}\right)\delta k_x. \tag{85}$$

Therefore, in the momentum representation, the generalized orbital angular momentum operator can be alternatively expressed as

$$\widetilde{L}_z = \frac{\tilde{v}_y}{v_x} x\delta k_y - \frac{v_x}{\tilde{v}_y} y\delta k_x = -i\frac{\partial}{\partial\phi}. \tag{86}$$

Thus the eigenstates of the generalized orbital angular momentum operator are exactly given by $\psi_l = \exp\left[il\phi(\delta\mathbf{k}_{xy})\right]$ with $l$ denoting the orbital angular quantum number:

$$\widetilde{L}_z \exp\left[il\phi(\delta\mathbf{k}_{xy})\right] = l\exp\left[il\phi(\delta\mathbf{k}_{xy})\right]. \tag{87}$$

In addition, the generalized total angular momentum operator is given by

$$\widetilde{J} = \widetilde{L}_z - \hat{S}_x, \tag{88}$$

where the negative sign in font of $\hat{S}_x$ is utilized for the sake of the consistent chirality of the spin frame $(\hat{S}_z, \hat{S}_y, -\hat{S}_x)$ and of the coordinate frame $(x, y, z)$. Then it can be directly verified that the generalized total angular momentum commutes with the Hamiltonian:

$$\left[\widetilde{J}, \hat{H}(\delta\mathbf{k}_{xy})\right] = 0. \tag{89}$$

Therefore, the generalized total angular momentum quantum number $j = l - s$ is conserved during the wave propagation along the $z$ direction. For an incident state with $l_i = 0$ orbital angular momentum and $s_i$ spin momentum, the output state satisfies $j = l_f - s_f = 0 - s_i$, thus the final orbital quantum number $l_f = s_f - s_i$ is determined by the difference of the finial and initial spin quantum numbers.

## Supplementary References

[1] Mostafazadeh, A. Pseudounitary operators and pseudounitary quantum dynamics. *J. Math. Phys.* **45**, 932–946 (2004).
[2] Binding, P. & Volkmer, H. Eigencurves for Two-Parameter Sturm-Liouville Equations. *SIAM Rev.* **38**, 27–48 (1996).
[3] Kutsenko, A. A., Shuvalov, A. L., Poncelet, O. & Norris, A. N. Spectral properties of a 2D scalar wave equation with 1D periodic coefficients: Application to shear horizontal elastic waves. *Math. Mech Solids* **18**, 677–700 (2013).
[4] Sakoda, K. Double Dirac cones in triangular-lattice metamaterials. *Opt. Express* **20**, 9925–9939 (2012).
[5] Hu, H., Hou, J., Zhang, F. & Zhang, C. Topological Triply Degenerate Points Induced by Spin-Tensor-Momentum Couplings. *Phys. Rev. Lett.* **120**, 240401 (2018).
[6] Tóth, T. A. Quadrupolar Ordering in Two-Dimensional Spin-One Systems (2011). Library Catalog: infoscience.epfl.ch.
